# Exercise and Childhood Cancer: From Science to Life “The 3rd Pediatric Exercise Oncology Congress Meets FORTEe”

**DOI:** 10.3390/curroncol33090524

**Published:** 2026-08-31

**Authors:** S. Nicole Culos-Reed, Miriam Götte, Jörg Faber, Sabine V. Kesting

**Affiliations:** 1 Faculty of Kinesiology, University of Calgary, Calgary, AB T2N 1N4, Canada; 2 Sports and Exercise Therapy, University Hospital Essen, 45147 Essen, Germany; miriam.goette@uk-essen.de; 3 Childhood Cancer Center, University Medical Center Mainz, 55131 Mainz, Germany; faber@uni-mainz.de; 4 Centre for Child and Adolescent Medicine, Technical University of Munich University Hospital, 81675 Munich, Germany; sabine.kesting@tum.de

**Keywords:** pediatric, adolescent, cancer, movement, exercise, physical activity

## Abstract

On behalf of the organizing committee, we are pleased to present the abstracts from the 2026 conference, “PEOC meets FORTEe”. The Third Pediatric Exercise Oncology Congress, this year meeting in person with the EU Study FORTEe (Get Strong to Fight Childhood Cancer: An Exercise Intervention for Children and Adolescents Undergoing Anti-Cancer Treatment), brought together scientists, clinicians, exercise professionals, physiotherapists, patients, families, community partners, and advocates from across the globe. Over two intensive days, 142 participants from 24 countries, guided by seven invited speakers, engaged in rigorous, wide-ranging, and interdisciplinary exchange that our field needs for sustaining growth and addressing key issues. The conference was held in Mainz, Germany, on 16–17 April 2026 and explored the growing field of evidence examining the impacts of exercise on basic biological mechanisms through to patient perspectives on quality of life. There was also a clear focus on knowledge translation, moving evidence to inform clinical integration of exercise to support beneficial outcomes across treatment trajectories. We would like to thank all of the researchers and presenters for their work and for contributing to the positive impact on patients and families who journey through a childhood or adolescent cancer diagnosis. The Congress Presidents and Scientific Committee Lead of the Third Pediatric Exercise Oncology Congress meets with FORTEe 2026. Scientific Committee co-leads included Miriam Götte, Nicole Culos-Reed, and Marie Neu. Scientific Committee members included Alejandro Lucía, Amanda Wurz, Carolina Chamorro Viña, Corina Rueegg, Elias Dreis-Mickenbecker, Emma Verwaaijen, Helen Griffith, Joachim Wiskemann, Jörg Faber, Maria Olsson, Maxime Caru, Nivedita S. Prabhu, Sara Grimshaw, and Vesile Yıldız Kabak. Congress organization was through the University Medical Center Mainz.

## Simple Summary

Pediatric exercise oncology examines the impact of exercise across the treatment journey and into survivorship. This includes examining potential biological mechanisms; physical, mental, and emotional well-being potential outcomes; and the implementation of exercise interventions across clinical and community settings. Despite the growing evidence on positive impacts, exercise largely remains separate from pediatric cancer care. The Pediatric Exercise Oncology Congress, in conjunction with the European research teams leading FORTEe (a Horizon 2020-funded study across 10 European countries), was held in Mainz, Germany, in April 2026. Two days of invited talks, research oral and poster presentations included research on biological mechanisms, clinical integration and modes of delivery, intervention impacts ranging across physical and psychosocial outcomes, and patient and community partner perspectives. The conference provided an opportunity to build networks, engage in discussions on what ‘really’ matters to grow the research and impact of this field, and the need to move evidence to practice to support wellness across childhood and adolescent cancer journeys.

## 1. Foreword by the Congress Presidents and Scientific Committee Lead

There are moments in science when a field reaches an inflection point—when the accumulated weight of evidence, the convergence of clinical practice, and the energy of an emerging community of researchers and practitioners tip toward transformation. We believe this congress represents precisely such a moment for pediatric exercise oncology.

The Third Pediatric Exercise Oncology Congress, this year meeting with the EU Study FORTEe, brought together an extraordinary gathering of scientists, clinicians, exercise professionals, physiotherapists, patients, families, and advocates from across the globe. Over two intensive days, 142 participants from 24 countries, guided by seven invited speakers, engaged in the kind of rigorous, wide-ranging, and genuinely interdisciplinary exchange that our field both needs and deserves. The result is this abstract volume, a document that captures the state of pediatric exercise oncology at an exciting juncture.

The timing could not have been more deliberate. FORTEe (Get Strong to Fight Childhood Cancer: An Exercise Intervention for Children and Adolescents Undergoing Anti-Cancer Treatment) is a landmark Horizon 2020-funded randomized controlled trial that enrolled 478 children, adolescents, and young adults with cancer across ten European centers. It is the largest exercise oncology trial ever conducted in a pediatric population undergoing cancer treatment, and its results represent a significant contribution to evidence-based pediatric oncology care.

Three keynote speakers set the congress tone. First, David Mizrahi reported results from the MERRIER study in Australia that demonstrated the feasibility and impact of telehealth-delivered exercise for children with cancer. Carmen Fiuza-Luces and Alejandro Lucia overviewed the biological mechanisms by which exercise modulates anticancer immunity, from the mobilization of natural killer cells and cytotoxic T lymphocytes to the reshaping of the tumor microenvironment. The message that exercise is not merely a supportive therapy for quality of life, but may be a direct biological agent acting on cancer itself, is compelling and driving new research. This was reinforced by Keri Schadler in her keynote, whose preclinical work in Ewing sarcoma, pancreatic cancer, and melanoma models demonstrated how moderate-intensity exercise can remodel tumor vasculature, reduce immunosuppressive cancer-associated fibroblasts, and rewire the metabolic signaling of tumor cells. For those who still wonder whether exercise “really” matters in oncology, these presentations provided compelling answers rooted in molecular biology.

This congress also had a strong focus on implementation and practice. There remains a gap between evidence guidelines and practice, despite the growing body of data supporting exercise as safe, feasible, and beneficial throughout the cancer continuum. Calls for harmonized, actionable, pediatric-specific exercise guidelines resonated across every session that followed. Integrating patients’ voices will help us address needs and challenges, which then inform implementation.

The scientific breadth represented in this volume is striking. Readers will find studies ranging from biological mechanisms to clinical application, from systematic reviews to implementation studies, and across methodological approaches. This is a reflection of the genuine methodological maturity that pediatric exercise oncology has reached.

The FORTEe trial itself generated multiple contributions beyond its primary outcomes. The ethical dimensions of conducting exercise RCTs with children, from the interpretation of assent to the burden of control group allocation on families and staff, were examined. The experiences of implementing exercise therapy ‘starting from zero’ in Ljubljana, Slovenia, where no prior infrastructure existed, offered a model for how trials can catalyze lasting systemic change. The perspectives of children, adolescents, and healthcare professionals on digital tools (augmented reality apps, motion tracking systems, telehealth platforms) added a crucial patient-centered layer to the technology debate.

We are also proud that this congress created space for global perspectives, including contributions from Brazil, Uruguay, India, the Czech Republic, Sweden, Turkey, South Africa, and beyond. This work reminds us that pediatric exercise oncology is a global endeavor, and that the field’s most important unfinished business lies precisely in the contexts that remain most under-resourced and underrepresented in the literature.

What emerges from reading these abstracts is both humbling and energizing. We are reminded how much work remains, that exercise is still not standard care in most pediatric oncology settings worldwide, that evidence-based guidelines remain insufficiently detailed for everyday clinical use, and that access is profoundly unequal. We also see the quality, diversity, and ambition of the work being conducted worldwide. One of the most pressing questions that remained unanswered at the end of the conference centers on exercise dose. Specifically, is there an ideal amount of exercise, and when is too much training counterproductive? It is clear that we must continue our research, work closely together, pool our findings and methods, and work together to fill the critical gaps in our knowledge to positive impact children and their families across the pediatric cancer journey.

We extend our deepest gratitude to all authors, presenters, reviewers, and participants who made this congress possible, and to the FORTEe consortium for the partnership that shaped its spirit. We dedicate this volume to the children and families who participated in these studies and programs whose engagement make our science possible.

**Invited Talk**
Effects of a Structured Exercise Intervention in Childhood Cancer Patients during Intensive Treatment: Results of the Multicenter Randomized Controlled FORTEe Trial
Marie A. Neu ^1^ and Elias Dreismickenbecker ^1^ on behalf of the FORTEe Consortium
^1^ Childhood Cancer Center Mainz, University Medical Center of the Johannes Gutenberg-University Mainz, Mainz, Germany

The FORTEe trial is a randomized, controlled, multicenter study evaluating a personalized and standardized exercise intervention in children, adolescents, and young adults undergoing cancer treatment across ten European centers. Given the persistent burden of treatment-related morbidity, including fatigue, physical inactivity, and reduced quality of life, the trial investigated the integration of structured exercise into pediatric oncology care. Participants were randomized to either an 8–10-week supervised exercise program or to usual care. The intervention combined endurance, strength, flexibility, and balance training, supported by digital and telehealth solutions to enable both in-person and remote participation. Overall, 478 patients (mean age 11.6 ± 4.4 years; 43% female) were enrolled, making FORTEe one of the largest exercise oncology trials conducted in pediatric cancer patients to date. The project aims to strengthen the evidence base for pediatric exercise oncology and support the implementation of exercise as a standard component of childhood cancer care worldwide.

**Invited Keynote**
Revolutionizing Recovery: The Power of Digital and Community-Based Exercise for Childhood Cancer Survivors
David Mizrahi ^1^
^1^ The Daffodil Centre, The University of Sydney, and Cancer Council NSW, Sydney, Australia

Survivors of childhood cancer often experience long-term physical, psychological, and social challenges resulting from treatment, including fatigue, reduced fitness, and decreased quality of life. Exercise and physical activity are increasingly recognized as essential components of survivorship care, yet access remains a significant barrier, particularly for families living in rural or regional areas or when treatment centers do not offer dedicated programs. Emerging digital health technologies and community-based partnerships provide new opportunities to bridge this gap and deliver accessible, sustainable models of care. This presentation will outline innovative approaches that integrate telehealth, digital tools, and community partnerships to support exercise participation among childhood cancer survivors. Recent qualitative research exploring families’ and clinicians’ perspectives highlights the potential of technology—including wearable activity trackers, gamified physical activity, and telehealth platforms—to enhance motivation and engagement in physical activity. Findings from the MERRIER study, a hybrid effectiveness-implementation study of a telehealth-delivered exercise program, are presented. This study demonstrates the feasibility and impact of remotely delivering structured, individualized exercise interventions, alongside strategies to evaluate program reach, adoption, implementation, and sustainability. The role of community organizations, such as Camp Quality, in supporting recruitment and education, and Little Big Steps, which funds physiotherapists to provide bedside exercise services and wearable devices during treatment, will also be discussed. By combining digital health innovation with strong community engagement, these models of care have the potential to improve accessibility, equity, and health outcomes for children and families, ultimately transforming the recovery journey for childhood cancer patients and survivors worldwide.

**Invited Keynote**
Exercise and Anticancer Immunity
Carmen Fiuza-Luces ^1^ and Alejandro Lucia ^2^
^1^ Physical Activity and Health Research Group, Research Institute of the Hospital 12 de Octubre, Madrid, Spain
^2^ Department of Sport Sciences, Faculty of Medicine, Health and Sports, Universidad Europea de Madrid, Madrid, Spain

A growing body of evidence supports the role of physical exercise as a protective factor against cancer development, progression, and recurrence. Epidemiological studies consistently demonstrate that individuals who engage in regular exercise exhibit lower incidence rates of several malignancies, as well as reduced cancer-related mortality. Complementary findings from preclinical research further reinforce this association, showing that exercise interventions can slow tumour growth and delay disease progression even in aggressive cancer models.

Despite these consistent observations, the biological mechanisms underlying the anticancer effects of exercise are not yet fully elucidated. One of the most compelling hypotheses involves the modulation of the immune system. Both acute and chronic exercise appear to influence immune surveillance by mobilizing and activating multiple immune cell populations, including natural killer cells, cytotoxic T cells, and other effectors involved in tumour recognition and elimination. Importantly, exercise-induced changes may enhance the ability of these cells to infiltrate tumour tissue and exert antitumour activity.

In addition to systemic immune adaptations, emerging evidence suggests that exercise may create a more favourable tumour microenvironment, potentially improving vascularisation, reducing inflammation, and facilitating immune cell access to malignant tissue. Moreover, recent experimental approaches indicate that immune cells obtained following an acute bout of exercise may have enhanced functional capacity, raising the possibility of leveraging exercise as an adjuvant strategy in adoptive cell therapies.

The presentation provided an overview of current knowledge regarding the relationship between physical activity and cancer, with particular emphasis on the differential effects of acute versus regular exercise on immune function. Mechanistic insights derived from both human and animal studies were discussed, and highlight the translational potential of integrating structured exercise interventions into oncology care. Understanding how exercise modulates anticancer immunity may open new avenues for improving patient outcomes and developing innovative therapeutic strategies.

**Invited Keynote**
Exercise Impacts the Tumor Microenvironment and Response to Therapy
Keri Schadler ^1^
^1^ Department of Pediatrics Research, University of Texas MD Anderson Cancer Center in Houston, TX, USA

Exercise for cancer patients and survivors is well known to confer benefits for quality of life. Preclinical research suggests that in addition to these benefits, exercise changes the tumor microenvironment in several ways that support improved efficacy of chemotherapy and immunotherapy: exercise improves tumor blood vessel function, reduces tumor-promoting fibroblasts, changes the infiltrating immune milieu, and regulates metabolic signaling in tumor cells. For example, two weeks of treadmill exercise at moderate intensity remodeled blood vessels in Ewing sarcoma in mice. This led to improved delivery of chemotherapy to the tumors, inhibiting tumor growth better than chemotherapy alone. In addition to improving the function of blood vessels, treadmill exercise decreased the number of inflammatory cancer-associated fibroblasts (CAFs) in mouse models of pancreatic cancer. Fewer inflammatory CAFs is associated with better drug and immune cell access to kill tumor cells, and with a phenotypic shift away from immune suppression toward activation within infiltrating immune cells. Finally, emerging evidence suggests that exercise rewires metabolic signaling in tumor cells. In mouse melanoma, two weeks of treadmill exercise suppressed glycolytic flux in tumor cells, favoring utilization of alternative fuel sources including fatty acids. Interestingly, in tumors from exercised mice, ATP reduction was impaired, and apoptosis was increased regardless of glycolytic state, suggesting that tumors are unable to keep up with energy demand. Taken together, there is evidence that exercise profoundly changes virtually every cell type within the tumor microenvironment, making exercise an appealing adjuvant to many cancer therapies.

## 2. Invited Symposia

### 2.1. Exercise Guidelines for Children and Adolescents with Cancer—From Evidence to Practice

Sabine Verena Kesting ^1^
^1^ Department of Pediatrics. TUM School of Medicine and Health, Technical University of Munich, Germany; German Center for Child and Adolescent Health (DZKJ), partner site Munich, Germany

Exercise is increasingly recognized as an essential component of modern pediatric oncology care. Children and adolescents with cancer are at high risk for physical inactivity, declining physical fitness, and acute as well as long-term treatment-related side effects. Although growing evidence demonstrates that exercise is safe, feasible, and beneficial during and after cancer treatment, translation into standardized clinical practice remains limited due to medical complexity, heterogeneity of diagnoses and treatments, safety concerns, and the lack of pediatric-specific standardization. Existing recommendations, including general physical activity guidelines for healthy children and adolescents, exercise guidelines for adult cancer survivors, and pediatric oncology recommendations such as the international Pediatric Oncology Exercise Guidelines (iPOEG) and the Network ActiveOncoKids consensus recommendations, provide important orientation but still lack sufficiently detailed and actionable guidance for routine pediatric oncological care. Current evidence supports the implementation of individualized and appropriately adapted exercise interventions to improve physical performance, quality of life, and long-term health outcomes in childhood cancer patients and survivors. Harmonized, clinically applicable exercise guidelines are needed to ensure safety, support clinical decision-making, maintain quality of care, and facilitate implementation across institutions. Ongoing international efforts, therefore, focus on developing more detailed exercise guidelines for children and adolescents with and beyond cancer. These approaches aim to translate evidence into practical recommendations for individualized exercise prescription and implementation in pediatric oncology and survivorship care.

### 2.2. Bridging Medicine and Movement—Integrating Exercise Therapy into Oncology Care

Irene von Luettichau ^1^ Katrin Burkhardt *
^1^ Department of Pediatrics. TUM School of Medicine and Health, Technical University of Munich, Germany; German Center for Child and Adolescent Health (DZKJ), partner site Munich, Germany
* Patient Representative

The integration of exercise therapy into pediatric oncology care is a promising approach to potentially improve physical and psychosocial aspects like functional capacity, muscle strength, anxiety and quality of life in cancer patients and survivors. Exercise can help preserve activities of daily living and autonomy during treatment, while promoting inclusion and social participation after completion of therapy. Mechanistically, evidence is increasing regarding the biological impact of exercise. Systemic effects through modulation of inflammation, immune surveillance, metabolic regulation, and psychosocial resilience have been and continue to be identified.

Despite substantial evidence supporting the benefits of structured exercise throughout the cancer continuum, exercise interventions remain insufficiently implemented in routine clinical practice. Successful integration of exercise therapy into acute and follow-up pediatric oncology care requires close collaboration among oncologists, physiotherapists, exercise professionals, nursing staff, and affected patients, survivors, and families. Individualized exercise prescriptions tailored to cancer type, treatment phase, comorbidities, and personal preferences are essential to ensure safety and adherence. However, implementation remains limited by insufficient institutional resources, inadequate professional training, lack of reimbursement structures, and low patient awareness.

Future oncology care models should establish exercise therapy as a standard supportive intervention alongside pharmacological and surgical treatments. Key priorities include developing evidence-based guidelines, strengthening interdisciplinary education, and expanding access to exercise to facilitate clinical implementation. Bridging medicine and movement offers a valuable opportunity to advance patient-centered oncology care, potentially improve long-term survivorship outcomes, and serve as a prevention approach for treatment-related sequelae.

### 2.3. Physiotherapist Perspective on Paediatric Exercise Oncology in Low- and Middle-Income Countries

Precious Madzimbe ^1,2^
^1^ Department of Health and Rehabilitation Sciences, Faculty of Health Sciences, University of Cape Town
^2^ Department of Physiotherapy and Occupational Therapy, United Bulawayo Hospitals

Childhood cancer survival has improved globally; however, many children continue to experience significant functional morbidity during and after treatment, including fatigue, muscle weakness, neuropathy, reduced aerobic capacity, deconditioning, and reduced participation in play and schooling. This invited presentation explored the role of physiotherapists in paediatric exercise oncology within low- and middle-income countries (LMICs), particularly in Africa. The presentation highlighted the substantial rehabilitation gap in LMICs, characterised by limited rehabilitation workforce capacity, delayed referral pathways, lack of structured exercise oncology programmes, inadequate infrastructure, and restricted access to equipment. Despite these challenges, growing evidence demonstrates that exercise interventions are safe and beneficial for children with cancer, improving muscle strength, cardiorespiratory fitness, fatigue, and quality of life.

The presentation discussed practical physiotherapist-led rehabilitation approaches suitable for low-resource settings, including family-centred rehabilitation, caregiver training, home-based programmes, community follow-up, and school reintegration. Examples of feasible low-cost interventions included theraband strengthening, play-based aerobic activities, walking circuits, and bed-based mobility exercises. Safety considerations, including platelet count precautions, neutropenia management, fatigue monitoring, and medical clearance prior to exercise prescription, were also emphasised. Furthermore, the presentation proposed a scalable LMIC paediatric exercise oncology model integrating oncology teams, physiotherapist-led exercise prescription, family involvement, and community-based rehabilitation. The presentation concluded that paediatric exercise oncology is feasible in African settings and should become an integrated component of routine oncology care pathways, with physiotherapists playing a central leadership role in implementation, advocacy, and future rehabilitation research.

## 3. Oral Research Presentations

### 3.1. Session 1—Physical Function from Diagnosis to Survivorship

#### Respiratory Patterns During Exercise in Children, Adolescents and Young Adults with Cancer

Lena Wypyrsczyk ^1^, Linda Peli ^2^, Wiliam Zardo ^3^, Tommaso P. Moriggi ^2^, Emanuele Villa ^2^, Eleonora Corti ^2^, Mareike Kühn ^1^, Elias Dreismickenbecker ^1^, Marie A. Neu ^1^, Alejandro Lucia ^4^, Carmen Fiuza-Luces ^5^, Eva Santa Cruz-Ramos ^5^, Filippo Spreafico ^3^, Alessandra Busia ^6^, Martin K. Fridh ^7^, Joachim Wiskemann ^8^, Adriana C. Balduzzi ^9,10^, Francesca Lanfranconi ^2^ and Joerg Faber ^1^ on behalf of the FORTEe consortium
^1^ University Medical Center of the Johannes Gutenberg-University Mainz, Childhood Cancer Center Mainz, Mainz, Germany
^2^ Fondazione Monza e Brianza per Il Bambino e La Sua Mamma, Centro Maria Letizia Verga, Monza, Italy
^3^ Fondazione IRCCS Istituto Nazionale dei Tumori, Pediatric Oncology Unit, Milan, Italy
^4^ Department of Sports Sciences, Faculty of Medicine, Health and Sports, Universidad Europea de Madrid, Madrid, Spain
^5^ Research Institute of the Hospital 12 de Octubre (‘imas12’), Madrid, Spain
^6^ Fondazione IRCCS Istituto Nazionale dei Tumori, Pneumology, Milan, Italy
^7^ Department of Pediatrics and Adolescent Medicine, Copenhagen University Hospital, Rigshospitalet, Copenhagen, Denmark
^8^ Working Group Exercise Oncology, Department of Medical Oncology, Heidelberg University Hospital, Medical Faculty Heidelberg, Heidelberg University, National Center for Tumor Diseases Heidelberg, a partnership between DKFZ and Heidelberg University Hospital, Heidelberg, Germany
^9^ Pediatric Department, Fondazione IRCCS San Gerardo dei Tintori
^10^ School of Medicine and Surgery, Milano-Bicocca University, Monza, Italy

**Introduction:** Pulmonary ventilation (VE) efficiency depends on the interaction of respiratory frequency (Rf) and tidal volume (Vt). Here, we define this interaction as respiratory patterns. Using cardiopulmonary exercise testing (CPET), this study aimed to characterize respiratory patterns in childhood cancer patients during the early phase of intensive treatment and examine their associations with peak oxygen uptake (VO2peak). **Methods:** A subsample of 67 childhood cancer patients (mean age: 13.7 ± 2.9 years; 65.7% male) from the FORTEe multicenter randomized controlled trial (NCT05289739) was analyzed. CPETs followed incremental cycle ergometer protocols (n = 49) or an adapted Yo-Yo intermittent recovery test (n = 18). VO2peak was compared to predicted normative values using paired *t*-tests. Respiratory patterns were classified as very good, good, or poor responders based on the VE–Vt response and Rf efficiency. ANCOVA, adjusted for age, sex, and BMI, assessed group differences in VO2peak. **Results:** Mean VO2peak was 24.21 ± 6.67 mL/kg/min, significantly lower than normative values (*p* < 0.001). Fifteen participants (22.4%) were classified as very good responders, showing high Vt and a two-phase VE-Vt pattern with efficient Vt adaptation until late exercise, when Rf compensation predominated. Nineteen (28.4%) were good responders, with lower Vt, a steeper initial slope and earlier transition to Rf dominance. Thirty-three (49.2%) were poor responders, demonstrating a linear VE-Vt pattern dominated by Rf with limited Vt contribution. ANCOVA revealed significant group differences in VO2peak (*p* = 0.002), with poor responders showing significantly lower values than the other groups. **Conclusions:** Childhood cancer patients show significantly reduced VO2peak at the beginning of intensive treatment. Nearly half of these patients demonstrate inefficient respiratory patterns relying mainly on increased Rf without sufficient Vt adaptation. Assessing and classifying respiratory patterns during CPET may provide a promising approach for future studies on risk stratification and individualized interventions in pediatric oncology.

This project has received funding from the European Union’s Horizon 2020 research and innovation programme, under grant agreement No 945153.

### 3.2. Exercise Intolerance and Return to Sports in Survivors of Pediatric Hematologic Malignancies

Linda Peli ^1^, Emanuele Villa ^1^, Tommaso P. Moriggi ^1^, Luca Pollastri ^1^, Eleonora Corti ^1^, Giorgia Radaelli ^1^, Marta Corti ^1^, Federico Paoletti ^1^, Elisa Inselvini ^1^, Momcilo Jankovic ^1^, Adriana C. Balduzzi ^2,3^ and Francesca Lanfranconi ^1^
^1^ Fondazione Monza e Brianza per il Bambino e la sua Mamma—Centro Maria Letizia Verga, Monza, Italy
^2^ Department of Medicine and Surgery, University of Milano-Bicocca, Milan, Italy
^3^ IRCCS San Gerardo dei Tintori Hospital, Monza, Italy

**Introduction:** Pediatric patients undergoing treatment for hematologic malignancies experience a marked decline in exercise capacity due to the disease itself, its treatments, and prolonged physical inactivity. Initiating a personalized exercise program (PEx) at the time of diagnosis may mitigate these effects, which often persist into survivorship. Accurate assessment of residual exercise tolerance is essential for tailoring interventions. **Methods:** Patients aged 9–21 years with blood cancers were assessed at the start of maintenance therapy or at the end of treatment. Evaluations included a maximal cardiopulmonary exercise test (CPET) and functional performance tests. Key variables analyzed were peak oxygen consumption (VO_2_peak) and muscle oxygen extraction using near-infrared spectroscopy (NIRS, HHb/isch). **Results:** Twenty-six participants (mean age: 14.5 years; 58% female) completed the assessments. Diagnoses included acute lymphoblastic leukemia, acute myeloid leukemia, and lymphomas (11% had undergone hematopoietic stem cell transplantation). At the time of evaluation, 42% were in the maintenance phase, and 58% were off therapy for less than 12 months. Compared to healthy controls, participants exhibited a 32% reduction in VO_2_peak (range: 48–94%) and a 36% reduction in HHb/isch (range: 25–100%). **Conclusions:** Exercise intolerance persists during early survivorship, with considerable individual variability. Early implementation of structured PEx programs may facilitate the reintegration of pediatric cancer survivors into daily life and physical activity, including sports participation.

## 4. Differences in Body Composition and Physical Activity Among Long-Term Childhood, Adolescent, and Young Adult Cancer Survivors with Prediabetes

Rusha Bhandari ^1^, Kara Lukas, Xinyi Du, Meagan Echevarria, Lanie Lindenfeld, Kyuwan Lee, F. Lennie Wong and Saro Armenian
^1^ 1500 E Duarte Rd, 91,010 Duarte, California, United States

**Introduction:** Childhood, adolescent, and young adult (CAYA) cancer survivors experience a high burden of late effects, including diabetes and sarcopenic obesity. The prediabetes stage represents a critical window during which to intervene. There are limited data regarding the relationship between body composition and prediabetes in CAYA survivors. **Methods:** This cross-sectional study enrolled 100 CAYA survivors (prediabetes = 40, controls = 60, frequency matched by age and sex) ≥ 2 years from completion of cancer-directed treatment. Diabetes risk was evaluated with fasting glucose and hemoglobin A1c. Measures of body composition included body mass index (BMI, kg/m^2^), waist circumference (WC, cm), and estimates of fat and fat-free mass from bioelectrical impedance analysis. Physical activity was self-reported using the International Physical Activity Questionnaire. Odds ratios (per one unit measure) adjusted for hematopoietic cell transplantation (HCT) status, dyslipidemia, sex, and age were calculated to compare differences in body composition between groups. **Results:** Average age at diagnosis was 14.4 y (SD = 7.0), and at the time of study was 36.7 y (SD = 11.0); 42% were male, 42% were Hispanic, and 41% were non-Hispanic White. The most common underlying cancer diagnoses were acute lymphoblastic leukemia (41%) and Hodgkin lymphoma (21%). The majority received steroids (71%), alkylator chemotherapy (78%), and radiation therapy (56%); 41% received HCT (prediabetic group: 62.5% vs. controls: 26.7%, *p* < 0.001). BMI was not statistically significantly different between groups; however, the prediabetic group reported significantly less physical activity than controls (2784 vs. 4587 MET-min/week, *p* = 0.029). In multivariable models, higher WC and measures of adiposity were associated with increased odds of prediabetes (WC: OR = 1.04 [95%CI = 1.01–1.08]; fat mass index [kg/m^2^]: OR = 1.16 [95%CI = 1.03–1.31]; visceral adipose tissue index [L/m^3^]: OR = 8.12 [95%CI = 1.36–48.57]). **Conclusions:** Among long-term CAYA cancer survivors, increased WC and adiposity are associated with risk of prediabetes. Targeted physical activity programs may mitigate the risk of prediabetes and its sequelae in this vulnerable patient population.

### From Evidence to Implementation—The BEPPO Randomized Controlled Trial as a Step Towards Exercise Becoming Standard Care in Pediatric Oncology

Miriam Götte ^1^, Ronja Beller, Lena Böhlke, Sabine Kesting, Irene von Luettichau, Meinolf Siepermann, Vivien Lösse, Thorsten Simon, Uta Dirksen, Simon Elmers, Gabriele Escherich, Elias Dreismickenbecker, Joerg Faber, Mirjam Dieckelmann, Jan-Henning Klusmann, Pablo Hernáiz Driever, Olivia Wegner, Petra Neunsinger, Jana Ernst, Anne Seime, Dominik Gaser, Claudia Blattmann, Lisa Nonnenmacher, Sebastian Viktor Waldemar Schulz, Gabriele Calaminus, Katja Baust, Reinhard Sablowski, Kerstin Rosenberger, Anna Hagemeier, Martin Hellmich, Hansjörg Baurecht, Christine Welker, Michael Leitzmann, Dusan Simic, Stephanie Stock and Freerk T. Baumann
^1^ West German Cancer Center, University Hospital Essen, University of Duisburg-Essen, Essen, Germany

**Introduction:** Exploratory studies indicate that exercise interventions during medical treatment improve physical performance and mental health in children and adolescents with cancer. However, exercise is not yet part of standard care. The main objective of the BEPPO study is to improve care for pediatric cancer patients by targeting mobility, fitness and symptom management to lower acute and long-term treatment burden. Consequently, the study seeks to generate a scientific evidence base to enable the implementation of exercise as an integral part of standard care. **Methods:** The BEPPO study is a Germany-wide, multicenter randomized controlled trial funded by the Innovation Fund of the German Federal Joint Committee (G-BA) for a period of 42 months. A total of N = 346 children and adolescents aged 3–18 years will be recruited across 13 pediatric oncology centers in Germany. Participants will be randomly assigned (1:1) to either an intervention group receiving the multimodal BEPPO exercise program or the control group receiving standard care at their local hospital. The intervention comprises a 6-month supervised exercise program with 3–5 sessions per week, which is carried out both in-hospital and via telemedicine at home, as well as four individual exercise counseling sessions. **Results:** The primary endpoint is leg extension strength; secondary outcomes include endurance, functional mobility, balance, physical activity, fatigue, quality of life, well-being, frailty, and treatment-related symptoms and side effects. A process evaluation will assess implementation fidelity and identify barriers and facilitators to intervention delivery. Collaboration with five statutory health insurance providers will enable a health economic evaluation of treatment costs. **Conclusions:** The BEPPO trial includes several innovative approaches to advance pediatric exercise oncology research: a long-term exercise intervention during active treatment, a randomized comparison to standard care, and a large, diverse cohort. Effectiveness, implementation and health economic evaluations will provide relevant information for sustainable implementation of exercise into pediatric oncology care.

## 5. Session 2—Insights from Novel Interventions and Assessments (Rapid Orals)

### 5.1. Compensatory Gait in Childhood Cancer? How Motor Impairments Shape Walking Patterns: A Cross-Sectional Correlation Study

Mareike Kühn ^1,2,3^, Lena Wypyrsczyk ^1,2,3^, Elias Dreismickenbecker ^1,2,3^, Marie A. Neu ^1,2^ and Joerg Faber ^1,2^
^1^ Paediatric Haematology/Oncology, Department of Paediatrics, University Medical Centre of the Johannes Gutenberg-University Mainz, Mainz, Germany
^2^ Childhood Cancer Centre Mainz, University Cancer Centre Mainz (UCT Mainz), University Medical Centre of the Johannes Gutenberg-University Mainz, Mainz, Germany
^3^ Johannes Gutenberg-University Mainz, Institute of Sport Science, Mainz, Germany

**Introduction:** Safe and unrestricted walking is essential for participation in everyday life. It depends on the balanced contribution of muscle strength and joint mobility, modulated by precise sensory feedback. In children with cancer, motor impairments like reduced strength, limited joint mobility, and balance deficits can disrupt this interplay and lead to compensatory gait adaptations. However, the relationship between gait and physical performance has not yet been systematically investigated. Therefore, this study aims to examine this relationship in childhood cancer patients. The findings may help identify potential compensatory strategies and support the development of targeted interventions to improve walking ability and reduce fall risk. **Methods:** Sixty-four childhood cancer patients and survivors aged 9.75 ± 4.01 years underwent gait analysis and physical performance assessments (lower limb strength, joint range of motion, balance, functional mobility). Performance data were compared with age- and sex-matched normative values using Wilcoxon signed-rank tests. Associations between physical performance and gait were analyzed using partial Spearman’s rank correlations, adjusted for age, height and weight. Significance was set at *p* < 0.05. **Results:** Participants showed significant deficits in gait (step length, base of support, walking speed; *p* < 0.01) and physical performance parameters (lower limb strength, joint mobility, balance, functional mobility; *p* < 0.05) compared to normative values. Correlation analysis revealed significant associations between strength, joint mobility, balance, functional mobility, and gait parameters (*p* < 0.05). **Conclusions:** Our findings highlight noticeable impairments in gait and physical performance in children and adolescents with cancer. Observed adaptations in gait patterns may reflect compensatory strategies in response to limitations in strength, joint mobility, or balance, but no causal relationships can be inferred. Regular assessments of gait and physical performance may help detect deficits early and guide personalized interventions to support safe ambulation and long-term functional outcomes.

### 5.2. Prehabilitative Exercise in Children and Adolescents with Soft Tissue and Bone Sarcoma of the Lower Extremity—A Feasibility Study

Jennifer Queisser ^1,2,3,4^, Irene Schmid ^5^, Renate Oberhoffer-Fritz ^1^, Klaus Wörtler ^6^, Irene von Luettichau ^2,3,4^ and Sabine Kesting ^2,3,4^
^1^ Technical University of Munich, Munich, Germany
^2^ Department of Pediatrics. TUM School of Medicine and Health, Technical University of Munich, Germany; German Center for Child and Adolescent Health (DZKJ), partner site Munich, Germany
^3^ Children’s Oncology Network Bavaria, Munich, Bavaria, Germany
^4^ Bavarian Cancer Research Center (BZKF), Munich, Germany
^5^ Dr. von Hauner Children’s Hospital, Ludwig-Maximilians-University Munich, Munich, Germany
^6^ Department of Radiology and Musculoskeletal Radiology Section, Klinikum Rechts der Isar, TUM School of Medicine, Technical University of Munich, Munich, Germany

**Introduction:** Soft tissue and bone sarcomas (SBSs) of the lower extremity pose substantial challenges for affected children and adolescents. Chemotherapy, surgery, and load restrictions lead to long-term physical limitations and reduced activity levels, compromising physiological development, resilience, and autonomy. Therapeutic exercise programs typically address these deficits post-operatively, while the evidence regarding pre-operative (“prehabilitative”) interventions remains unclear. Therefore, this study investigates the feasibility of a supervised, prehabilitative training program and gathers preliminary data on its potential to optimize pre-operative condition and post-operative recovery. **Methods:** This bicentric study, designed as a controlled clinical trial, enrolls patients (6–18 years) with newly diagnosed primary SBS of the lower extremity. The recruitment period is from October 2024 to August 2026. Participants are allocated to either the intervention group (IG) or control group (CG), with a target sample size of n = 16–18. The IG participates in specific strength and balance exercises at least twice weekly for a minimum of 30 min per session during neoadjuvant therapy. The CG receives standard care without any exercise intervention. **Results:** Primary outcomes assess feasibility through recruitment, acceptance, data quality, practicability, and safety (adverse events). Secondary outcomes compare structural and functional measures—psoas muscle area, body composition, muscle strength, mobility, balance, gait, physical activity, and quality of life—at four time points from baseline at diagnosis to follow-up at one year post-treatment. Clinical course metrics (e.g., hospitalization duration, infection rates) are recorded as additional outcomes. Since data collection is ongoing, preliminary findings were presented at the congress. **Conclusions:** This study evaluates the feasibility of prehabilitative training in patients with SBS of the lower extremity and provides preliminary data on its effects in mitigating muscle loss, supporting physiological body composition, and improving functional outcomes. Enhancing daily functionality and autonomy significantly improves quality of life, highlighting the importance of investigating and promoting structured exercise interventions in this population.

### 5.3. Telemedicine-Based Adapted Physical Activity in Pediatric Oncology: Feasibility and Effectiveness

Laura Pianta ^1^, Linda Peli ^2^, Elisa Inselvini ^2^, Marta Cogliati ^1^, Eleonora Finazzi ^2^, Chiara Gorio ^3^, Alessia Angeloni ^2^, Joel Pollet ^1,4^, Richard F. Shumacher ^3^, Fulvio Porta ^3^, Filippo Bertoni ^2^ and Massimiliano Gobbo ^1,4^
^1^ Università degli Studi di Brescia, Brescia, Italy
^2^ Lega Italiana Lotta Tumori (LILT), Brescia, Italy
^3^ Azienda Socio Sanitaria Territoriale degli Spedali Civili di Brescia, Brescia, Italy
^4^ IRCCS Fondazione Don Carlo Gnocchi, Milan, Italy

**Introduction:** The number of children and young adults with cancer (CAYA-Cs) is increasing, making it one of the leading causes of pediatric mortality with physical and psychological side effects. Aims: To evaluate the feasibility and effectiveness of a telemedicine-integrated adapted physical activity (APA) program in pediatric oncology patients. **Methods:** CAYA-Cs aged 5–18 years participated in three weekly APA sessions tailored to their clinical condition. The intervention was delivered both in hospital rooms and remotely via Cisco Webex. The project enrolled 35 CAYA-Cs during pharmacological treatments, with a cut-off (>40%) established to evaluate the impact of telemedicine. The following tests were used to assess strength and mobility: handgrip (HG), sit-to-stand (STS), deltoid strength measured with dynamometer (DD), and chair sit-and-reach (CSR). Cardiorespiratory capacity and gait quality were assessed with IMU using the six-minute walking test (6MWT) and the timed up-and-go (TUG). **Results:** Fourteen CAYA-Cs (mean age: 10.11; 64% males) were included. Adherence to the training sessions had a mean of 76% (mean 71% for telemedicine). Diagnoses included 78% hematologic malignancies, 22% solid tumors, of whom 46% transplanted. The mean time from diagnosis to study enrollment was 198 days (12–1157). No relevant adverse events were observed during the evaluations. The following results were observed: HG (right: *p* = 0.006, left: *p* = 0.0416), STS (*p* = 0.0059), DD (right: *p* = 0.0182, left: *p* = 0.0426), and CSR (right: *p* = 0.41, left: *p* = 0.40). Gait analysis showed: 6MWT distance (*p* = 0.0409), average speed (*p* = 0.0428), average cadence (*p* = 0.0170), and stride length (*p* = 0.0079) improved; TUG duration (*p* = 0.0148). **Conclusions:** Telemedicine-based adapted physical activity appears feasible and effective for improving functional capacity in pediatric oncology patients, supporting integration of digital health solutions into long-term care.

### 5.4. Assessment of Muscle Strength, Sensory Functions, Motor Performance, Fatigue, and Quality of Life Level in Children with Acute Lymphoblastic Leukemia: A Prospective Follow-Up Study

Vesile Yıldız Kabak ^1^, Arda Oter ^1^, Selin Aytac ^2^ and Tulin Duger ^1^
^1^ Faculty of Physical Therapy and Rehabilitation, Hacettepe University, Ankara, Turkiye
^2^ Department of Pediatric Hematology, Faculty of Medicine, Hacettepe University, Ankara, Turkiye

**Introduction:** The aim of the present study was to examine muscle strength, sensory functions, motor performance, fatigue, and quality of life level in children with acute lymphoblastic leukemia (ALL) at diagnosis and at the end of induction chemotherapy. **Methods:** Children diagnosed with ALL at Hacettepe University in Ankara/Turkiye were included. Participants were assessed in their first week of hospital admission for induction chemotherapy and 2 to 3 days before discharge. Muscle strength (hip flexion, knee extension, and grip strength) was assessed using a hand-held dinamometer. Sensory functions were evaluated using the Pediatric-Modified Total Neuropathy Scale. The Bruninsky–Oseretsky Motor Proficiency Test 2nd Version-Short Form was used to evaluate motor performance. The fatigue and quality of life levels were assessed using the child report of the PedsQL Multidimensional Fatigue Scale and the child report of the PedsQL Cancer Module, respectively. **Results:** A total of six children were included. The mean age of the children was 12.50 ± 3.88 years. There was a statistically significant reduction in hip flexion muscle strength between the time points (106.10 ± 25.36 versus 72.96 ± 22.53 kg-f, *p* = 0.043). The fatigue level was also significantly higher at the end of induction therapy than baseline (81.24 ± 17.42 versus 55.83 ± 31.67 points, *p* = 0.041). The quality of life score was significantly lower at the end of treatment than at baseline (84.45 ± 13.35 versus 65.89 ± 12.45 points, *p* = 0.048). There was deterioration in the other outcome variables, but no significant difference was found (*p* > 0.05). **Conclusions:** The findings of this study suggest the necessity of exercise and physical activity interventions from the diagnosis in children with ALL, with the aim of reducing fatigue and enhancing muscle strength and quality of life. As this is an ongoing research study, a larger sample size is required to ensure the study’s statistical power.

### 5.5. The Use of Motion Tracking Technology to Support an Exercise Intervention for Children and Young People with Cancer: Perspectives of Users and Healthcare Professionals in the FORTEe Trial

Hayley Marriott ^1,^*, Alba Solera-Sanchez ^1,^*, Stanley Windsor ^1^, Kim Straun ^3^, Marie Neu ^4^, Elias Dreismickenbecker ^4^, Francesca Lanfranconi ^5^, Linda Peli ^5^, Joachim Wiskemann ^6^, Nikolai Bauer ^6^, Filippo Spreafico ^7^, William Zardo ^7^, Tobias Baader ^8^, Peter Wright ^1^, Joerg Faber ^4,^** and Eila Watson ^2,^** on behalf of the FORTEe Consortium
^1^ School of Sport, Nutrition and Allied Health Professions, Faculty of Health Science, and Technology Oxford Brookes University, Oxford, UK
^2^ Oxford Institute of Applied Health Research, Faculty of Health Science, and Technology, Oxford Brookes University, Oxford, UK
^3^ School for Policy Studies, University of Bristol, UK
^4^ University Medical Center of the Johannes Gutenberg-University Mainz, Childhood Cancer Center Mainz, Mainz, Germany
^5^ Fondazione Monza e Brianza per Il Bambino e La Sua Mamma, Monza, Italy
^6^ Working Group Exercise Oncology, Department of Medical Oncology, Heidelberg University Hospital, Medical Faculty Heidelberg, Heidelberg University, National Center for Tumor Diseases Heidelberg, a partnership between DKFZ and Heidelberg University Hospital, Heidelberg, Germany
^7^ Pediatric Oncology Unit, Fondazione IRCCS Istituto Nazionale dei Tumori, Milan, Italy
^8^ Pixformance Sports GmbH, Dallgow-Döberitz, Germany
* these authors share first authorship
** these authors share last authorship

**Introduction:** Technological advances offer new and creative approaches to help optimise oncology care. Previous research suggests that supportive technological tools can be helpful for children with cancer, and that using technology within exercise-based interventions may contribute to benefits such as higher physical activity levels and reduced cancer-related fatigue. This study explored the perspectives of children, adolescents and young adults with cancer (CAYAs) and exercise and healthcare professionals (EHCPs) on the use of an innovative motion tracking device (*Pixformance*) within the FORTEe clinical trial. **Methods:** CAYAs (aged 4–21 years) and EHCPs from five participating centres were eligible. The device, designed to support in-hospital training within an individualised exercise programme, featured an inbuilt camera system, avatar-led exercise demonstrations, and real-time feedback on exercise form. Half-structured interviews were conducted with CAYAs, and an anonymous online survey was completed by EHCPs. **Results:** A total of 90 CAYAs participated (mean age 11.1 years ± 3.7, 44.4% females) in the half-structured interview and 33 EHCPs in the online survey. CAYAs described the device as novel and helpful, particularly for ward-based exercise. They appreciated the variety of exercises available and found the exercise demonstrations helpful in enabling them to perform exercises correctly, though some technical issues limited usability. EHCPs reported benefits for supervised training but identified challenges related to transportability and offline functionality. Suggested improvements from both groups included adding gamification elements such as rewards, avatar customisation, and competitive features. **Conclusions:** The motion tracking device was well received by both CAYAs with cancer and the EHCPs, and the study suggests this type of technology could be a useful adjunct to traditional methods. Insights from this study highlight opportunities to optimise such technology to improve motivation, usability, and integration into clinical settings.

## 6. Session 3—Exercise and Implementation Needs (Rapid Orals)

### 6.1. Physical Activity and Sedentary Behavior Among Children in Sweden Who Survived a Brain Tumor

Åsa Persson Dobruna ^1^, Helena Igelström, Pernilla Åsenlöf, Gustaf Ljungman and Sara Frygner-Holm
^1^ Uppsala University Sweden, Uppsala, Sweden

**Introduction:** The majority of children who survive a brain tumor suffer from late complications. Physical activity can improve fitness and may mitigate some of the late complications. Previous studies show that childhood cancer survivors are less physically active than their peers. The aim of the study is to explore physical activity and sedentary behavior among children in Sweden after completing brain tumor treatment, and to examine the associations between late effects, physical activity, and sedentary behavior. **Methods:** A cross-sectional design using a survey containing questions about children’s physical activity, sedentary behavior, and late effects was implemented. A smaller subset had an activity tracker for 7 days. All children in Sweden aged 6–16 years who had finished brain tumor treatment six months to 10 years ago and their legal guardians were asked to participate in the study. **Results:** In total, 113 children (56 boys and 57 girls) and 123 legal guardians have responded so far; the data collection will end in October 2025. Of the respondents, 18% of children aged 6–12 meet the WHO physical activity recommendations, and 8.5% of children aged 13–16 meet them. Results on whether physical activity and sedentary behavior differed between brain tumor diagnoses/tumor locations, age groups, and sex were presented at the conference; likewise, whether any late complication affected physical activity and sedentary behavior according to the participants. **Conclusions:** The study is expected to provide new insights into physical activity and sedentary behavior among children who survived a brain tumor.

### 6.2. The Impact of the First Month After Diagnosis on Physical Activity, Sleep, Fatigue and Quality of Life in Children with Cancer

William Zardo ^1,2,3^, Letizia Galasso ^2^, Lucia Castelli ^2^, Andrea Ciorciari ^3^, Paolo Grampa ^1^, Maura Massimino ^1^, Angela Montaruli ^2^, Eliana Roveda ^2^ and Filippo Spreafico ^1^
^1^ Paediatric Oncology Unit, Fondazione IRCCS Istituto Nazionale dei Tumori, Milan, Italy
^2^ Department of Biomedical Sciences for Health, University of Milan, Italy
^3^ Department of Psychology, Università Cattolica del Sacro Cuore, Milan, Italy
^4^ Faculty of Education, Free University of Bozen-Bolzano, Brixen-Bressanone, BZ, Italy

**Introduction:** Acute and long-term morbidity related to childhood cancer and its treatment is concerning for the impact on patients’ quality of life (QoL). Precision-based exercise (PEx) is an effective strategy to enhance cardiorespiratory fitness and muscle strength, which are often impaired due to cancer or prolonged bed rest in the hospital. Additionally, structured exercise programs provide emotional and social benefits. Cancer-related fatigue (CRF) and sleep disturbances may further limit patient participation in PEx and sport programs. This study aims to assess physical activity (PA), sleep quality (SQ), CRF, and QoL within the first month after a pediatric cancer diagnosis. **Methods:** We interviewed a cohort of consecutive newly diagnosed participants (and their parents) using the Physical Activity Questionnaire for Adolescents (PAQ-A), Pittsburgh Sleep Quality Index (PSQI), Multidimensional Fatigue Scale (PedsQL-MFS), and Pediatric Quality of Life Inventory (PedsQL) at diagnosis (T0) and 30 days later (T1), when chemotherapy or radiation therapy had already been initiated. **Results:** We studied 12 patients with solid tumors (58% female, median age 16.8 ± 1.6 years, age range 14–19). When comparing T0 vs. T1, we observed significant reductions in PA (*p* = 0.041) and SQ (*p* = 0.001) and increased parents’ perception of CRF (*p* = 0.032) and adolescents’ CRF (*p* = 0.002). No significant changes were found in parents’ perception of QoL (*p* = 0.140) or adolescents’ QoL (*p* = 0.006). A better sleep quality was significantly correlated with PA (*p* = 0.001; *r*2: 0.27), CRF (*p* = 0.001; *r*2: 0.68), and QoL (*p* = 0.001; *r*2: 0.42). **Conclusions:** A pediatric cancer diagnosis has an immediate impact on levels of PA, SQ, CRF, and QoL—all variables affected at T0 due to cancer symptoms. These findings highlight the importance of early PEx development aimed at improving these aspects—hopefully to improve PA, SQ and QoL, while reducing fatigue. These strategies could serve as effective strategies to enhance outcomes in childhood cancer.

### 6.3. From Zero to Integration: Implementing Exercise Therapy in Pediatric Oncology Through the FORTEe Trial at Ljubljana Children’s Hospital, Slovenia

Milica Stefanović ^1^, Barbara Faganel Kotnik ^1^, Domen Ravnik ^1^, Meta Rovan ^1^, Barbara Konda ^2^, Marie A. Neu ^3^, Elias Dreismickenbecker ^3^, Mareike Kühn ^3^, Lena Wypyrsczyk ^3^, Francesca Lanfranconi ^4^, Adriana Balduzzi ^4,5^, Eila Watson ^6^, Hayley Marriott ^7^, Joachim Wiskemann ^8^, Nikolai Bauer ^8^, Rodolf Mongondry ^9^, Martin Kaj Fridh ^10^, Alejandro Lucia ^11^, Carmen Fiuza-Luces ^12^, Miriam Götte ^13^, Filippo Spreafico ^14^, Barbara Heißerer ^15^, Jörg Faber ^3^ and Lidija Kitanovski ^1^
^1^ University Medical Center Ljubljana, Ljubljana, Slovenia
^2^ Forma 3D Ltd., Ljubljana, Slovenia
^3^ University Medical Center of the Johannes Gutenberg-University Mainz, Childhood Cancer Center Mainz, Mainz, Germany
^4^ Fondazione Monza e Brianza per Il Bambino e La Sua Mamma, Centro Maria Letizia Verga, Monza, Italy
^5^ Fondazione IRCCS San Gerardo dei Tintori and School of Medicine and Surgery, Milano-Bicocca University, Monza, Italy
^6^ Oxford Brookes University, Oxford Institute of Applied Health Research, Oxford, UK
^7^ Oxford Brookes University, Oxford, UK
^8^ Heidelberg University Hospital, Working Group Exercise Oncology, Heidelberg, Germany
^9^ Centre de Lutte Contre le Cancer Léon Bérard, Lyon, France
^10^ University Hospital Copenhagen—Rigshospitalet, Copenhagen, Denmark
^11^ Universidad Europea de Madrid, Madrid, Spain
^12^ Research Institute of the Hospital, Madrid, Spain
^13^ University Hospital Essen, West German Cancer Center, Essen, Germany
^14^ Fondazione IRCCS Istituto Nazionale dei Tumori, Milan, Italy
^15^ Concentris Research Management, Fürstenfeldbruck, Germany

**Introduction:** Exercise therapy is increasingly recognized as a beneficial supportive intervention during cancer treatment. Within the Horizon 2020-funded FORTEe project, a randomized, controlled, multicenter trial in pediatric oncology using a personalized 8–10-week supervised exercise program is evaluated. At the University Medical Center Ljubljana (UKCL), no exercise therapy program existed before FORTEe. This made the site a unique setting for establishing exercise therapy from the ground up. **Methods:** Implementation was carried out jointly by UKCL and the consortium partner Forma 3D, under the supervision of the FORTEe coordinating site and relevant work package leads. Key challenges included the absence of a training room, the need to train non-study staff (oncologists, nurses) in exercise-related safety and clearance, and overcoming regulatory barriers. Two kinesiologists were recruited and trained by Forma 3D, and with political support and regulatory approval, were formally recognized as hospital-based healthcare professionals. **Results:** The site successfully embedded exercise therapy into daily pediatric oncology care despite the absence of pre-existing structures. All ward staff were trained to support exercise implementation, ensuring patient safety. Kinesiologists became integral members of the care team, and a sustainable structure was created through the employment of a full-time kinesiologist at the ward. With the support of the parents’ association *Junaki 3. nadstropja*, a project for establishing a dedicated exercise room has been prepared, and permits as well as financing have been secured to ensure long-term access to exercise therapy. **Conclusions:** Ljubljana’s participation in FORTEe demonstrates how a trial can catalyze the introduction of exercise therapy in a setting without any established exercise therapy framework. Success was enabled by clinical–industry collaboration, interprofessional training, regulatory innovation and parental support. This experience not only secured lasting change at UKCL but may also inform implementation strategies in other centers where exercise oncology structures are absent.

### 6.4. Physical Activity in an Oncology Outpatient Clinic: Experience and Perceptions from ATIVAONCO Project

Micheli Carminatti ^1^, Herber Orlando Benitez ^1^, Laís Nunes dos Santos ^2^, Érica Cappellaro ^2^, Júlia de Lima Ramos ^2^, Eduarda Eugênia Dias de Jesus ^1^ and Cíntia de la Rocha Freitas ^1^
^1^ Federal University of Santa Catarina, Florianopolis, Brazil
^2^ Federal University of Santa Catarina, Campus Universitário Trindade, Florianopolis, Brazil

**Introduction:** Childhood cancer affects not only physical health but also the psychosocial well-being of patients and their families. In this context, strategies that integrate physical activity into the hospital setting can transform the treatment experience by promoting quality of life and socialization. This study aimed to describe the implementation of the ATIVAONCO Project, a physical activity intervention program in a pediatric oncology outpatient clinic, and to analyze its effects on the dynamics of the environment and participant perceptions. **Methods:** This was an observational, qualitative, and descriptive study conducted at the Pediatric Oncology Outpatient Clinic of the Joana de Gusmão Children’s Hospital (Southern Brazil). Physical activity sessions were held twice a week, lasting 120 min each, and involved an average of 8 to 12 children per session. The activities were playfully designed and adapted, including health education moments with parents/caregivers, group stretching, and guided games/play for patients. Project evaluation was carried out through systematic observations, verbal feedback from children, and caregiver reports, which were then analyzed using content analysis. **Results:** From 2023 to the present, more than 250 children have participated in the sessions, highlighting the project’s consolidation and relevance. The activities transformed the waiting room into an active and welcoming space, fostering interaction and socialization. Children’s perceptions were broadly positive, with satisfaction reported in over 90% of the sessions evaluated. Caregivers emphasized improvements in mood, greater energy and engagement of their children, and recognized the humanizing role of the intervention within the outpatient setting. **Conclusions:** The ATIVAONCO Project demonstrated feasibility and a positive impact on hospital routines, modifying the outpatient environment and promoting physical, emotional, and social benefits. The high acceptance among participants reinforces the importance of incorporating Physical Education professionals into multidisciplinary pediatric oncology teams, with potential for replication in other healthcare contexts.

### 6.5. Exploring Links Between Parent and Child Physical Activity and Quality of Life in Pediatric Brain Tumor Patients and Survivors

Ariane Levesque PhD ^1,2^, Deepika Pugalenthi Saravanan MD student ^1^, Sebastien Perreault MD ^3^, Serge Sultan PhD ^4,5,6^, Leandra Desjardins PhD ^4^, Émélie Rondeau MS ^4^, Amanda Wurz PhD ^7^, Laurianne Buron PhD student ^4,5^, Nidhi B. Shah MD ^1^, Michael Hayes PhD ^8^, Lucia Romo PhD ^9,10^, Daniel Curnier PhD ^4,11^, Laurence Kern PhD ^12^ and Maxime Caru PhD ^1,13^
^1^ Department of Pediatrics, Penn State College of Medicine
^2^ Department of Neuroscience and Experimental Therapeutics, Penn State College of Medicine
^3^ Department of Neuroscience, University of Montreal
^4^ Research Center, Sainte-Jus>ne University Health Center
^5^ Department of Psychology, University of Montreal
^6^ Department of Pediatrics, University of Montreal
^7^ School of Kinesiology, University of the Fraser Valley
^8^ Department of Psychiatry and Behavioral Health, Penn State College of Medicine
^9^ Department of Psychology, University of Paris-Nanterre
^10^ Center for Epidemiology and Population Health, University of Versailles Saint-Quentin-en-Yvelines
^11^ School of Kinesiology and Physical Activity Sciences, University of Montreal
^12^ Ecole de Psychologues Praticiens, Lyon, Auvergne-Rhône-Alpes
^13^ Department of Public Health Sciences, Penn State College of Medicine

**Introduction**: This study examined how physical activity (PA) levels in parents and their children relate to children’s overall and domain-specific quality of life (QoL) among pediatric brain tumor (PBT) patients currently receiving treatment or in remission. **Methods**: In this cross-sectional study, 36 parent–child dyads reported their weekly minutes of PA. Children’s overall and domain-specific QoL (physical, emotional, social, and school functioning) were assessed using the PedsQL Generic Core Scales. Descriptive and correlational analyses explored associations between parent and child PA, and an Actor–Partner Interdependence Model (APIM) was used to assess how each individual’s PA contributed to the child’s QoL. **Results**: Parent and child PA levels were strongly and positively correlated (r = 0.802, *p* < 0.001). The APIM showed excellent model fit indices (χ2(8) = 3.40, *p* = 0.907, CFI = 1.000, TLI = 1.208, RMSEA = 0 (90%CI [0, 0.079]), SRMR = 0.077). A significant actor effect emerged, showing that higher child PA was associated with better overall QoL (β = 0.779, FDR-corrected *p* = 0.05, 95%CI [0.019, 0.108]). No partner effect was observed, as parent PA did not significantly predict child QoL (median FDR-corrected *p* = 0.290). **Conclusions**: While parents’ PA was not directly related to their child’s QoL, the strong association between parent and child PA behaviors highlights a potential indirect pathway to improve child QoL. Interventions that engage both parents and children in physical activity may foster increased child PA, which could in turn promote a better QoL in children diagnosed with a PBT. These findings emphasize the importance of developing family-centered approaches to promoting PA and QoL in pediatric oncology populations.

## 7. Session 4—Effective Intervention Strategies and Implementation Approaches

### 7.1. Digital Physical Activity Support for Childhood Cancer Survivors: Intervention Trial Results and Factors Affecting Real-World Implementation

Lauren Ha ^1,2,5^, Callum Joyner ^1,2,3^, Kiarn Roughley ^3^, Joseph Elias ^4^, Karen Johnston ^2^, Rachael Baldwin ^2^, Amanda Wurz ^5^, Natalie Taylor ^4^, Claire E. Wakefield ^1,6^ and Christina Signorelli ^1,2^
^1^ Discipline of Paediatrics & Child Health, School of Clinical Medicine, UNSW Sydney, Australia
^2^ Kids Cancer Centre, Sydney Children’s Hospital, Australia
^3^ Making Moves Youth Advisory Board
^4^ i2i, School of Population Health, UNSW Sydney, Australia
^5^ School of Kinesiology, University of the Fraser Valley, Canada
^6^ Department of Pediatrics, Stanford University and Stanford Medicine Children’s Health, Palo Alto, California, USA

**Introduction:** Engaging in regular physical activity may reduce cardiovascular risk, yet most childhood cancer survivors are insufficiently active and exercise support programs are not routine practice in Australia. The aims of this study were to evaluate a digital physical activity intervention called ‘Making Moves’ and simultaneously explore factors affecting its implementation. **Methods:** We conducted a type-1 hybrid effectiveness-implementation study. To evaluate effectiveness pre-/post-trial, we recruited survivors aged 8–21 years to complete Making Moves, an 8-week program with eight educational modules and five telehealth exercise physiology consultations. We conducted assessments of physical activity self-efficacy, depressive symptoms, exercise enjoyment, health-related quality of life (HRQL), aerobic fitness, and muscular endurance pre-/post-trial and at 6-month follow-up. To explore implementation, we conducted interviews with potential implementers using the Consolidated Framework for Implementation Research 2.0. **Results:** Thirty-three survivors enrolled, five dropped out and 26 completed the intervention (retention rate 85%). Survivors were aged 12.0 ± 3.4 years and 39% were female. No intervention-related adverse events were reported. Survivors’ mean physical activity self-efficacy scores (mean change = +5.6, *p* < 0.01) and aerobic fitness levels (mean change = +131 m, *p* < 0.05) significantly increased post-trial. No significant changes in muscular endurance, depressive symptoms, exercise enjoyment or HRQL were observed. Regarding implementation, 21 participants from community organisations (n = 3) and hospital clinics (n = 2) completed interviews. Beyond funding and resource constraints, additional barriers included limited program assistance and challenges related to language and cultural differences. Facilitators included positive attitudes toward implementing a physical activity program and the online modality of Making Moves. **Conclusions:** Making Moves has potential to improve survivors’ health outcomes. Sustained effects will be assessed at 6-month follow-up. Despite limited organizational funding, strong support for digital exercise services suggests a favourable context for scale-up. Strategies are needed to secure funding and embed physical activity support into paediatric survivorship care.

### 7.2. Interim Adoption and Implementation Results from a Videoconference-Delivered Physical Activity Intervention for Pediatric Cancer and Blood Disorder Patients in Alberta, Canada

Emma McLaughlin ^1^, S. Nicole Culos-Reed ^1^, Lauren Ha ^2^, Georgia Kaluznick ^1^, Gregory M. T. Guilcher ^3^, Beverly Wilson ^4^, Sara Fisher ^5^, Carolina Chamorro-Viña ^6^, In memory of Mira Penney ^7^, Bridget Penney ^7^, Janine Wich ^7^, Laura Wich ^7^ and Amanda Wurz ^8^
^1^ Faculty of Kinesiology, University of Calgary, 2500 University Dr NW, T2N 1N4 Calgary, Canada
^2^ School of Paediatrics and Child Health, School of Clinical Medicine, University of New South Wales, Sydney, Australia
^3^ Department of Pediatrics, Cumming School of Medicine, University of Calgary, Calgary, Canada
^4^ Department of Pediatrics, University of Alberta, Edmonton, Canada
^5^ Stollery Children’s Hospital, Edmonton, Canada
^6^ Kids Cancer Care Foundation and University of Calgary, Calgary, Canada
^7^ Participant Advisory Board, Canada
^8^ School of Kinesiology, University of the Fraser Valley, Chilliwack, Canada

**Introduction:** As evidence supporting physical activity (PA) for children with cancer continues to grow, so to have efforts to adopt and implement PA into standard care. Despite this, few studies have reported specific aspects of adoption and implementation. To address this gap, we aim to report interim adoption and implementation data from a trial evaluating a 12-week videoconference-delivered PA intervention for pediatric cancer and blood disorder patients in Alberta, Canada. **Methods:** Guided by the RE-AIM framework, adoption (i.e., number and sources of referrals) and implementation (i.e., trial retention, adherence to PA sessions, PA session fidelity, adverse events, intervention delivery time and cost) were tracked throughout trial delivery. Data were analyzed descriptively. Intervention delivery cost was calculated by multiplying base rate estimates for exercise professionals ($24) by delivery hours. Intervention/trial delivery coordination cost was calculated by summing salaries/stipends for staff. **Results:** Between March 2022 and July 2025, 98 referrals were received (89 = healthcare provider-referral; Alberta Children’s Hospital [n = 40], Stollery Children’s Hospital [n = 49], n = 9 self-referral). Implementation metrics indicated poor trial retention (completion of assessments) at 33%. Adherence to PA sessions was on average at 57% (i.e., 2 ± 1 sessions attended of 3 offered). PA session fidelity (intervention delivered as intended) was high at 95.2 ± 3.83%. No adverse events were reported. There were 204 PA session hours totaling $4900 ($350/participant) and 455 trial/intervention coordination hours, totaling $140,099.41 in salaries/stipends. **Conclusions:** We believe reporting adoption and implementation metrics can enhance transparency and enable more comprehensive intervention evaluation. Moving forward, we propose a core set of reporting metrics including number and sources of referrals, trial retention, adherence to PA sessions, PA fidelity, adverse events, and intervention delivery time and cost (overall and per-participant) to facilitate cross-study comparisons and support future PA implementation efforts in pediatric oncology.

### 7.3. Exercise Intervention for Patients with Adolescent Cancer: Preliminary Results of a Randomised Controlled Trial

Rodrigo Yagüe-Peñuelas ^1^, Susana Moreno-Acha ^2^, Belén Puente-Gómez ^3^, Javier Blanco-Elvira ^3^, Andrea Sierra-Ramos ^2^, Marta Iglesias-Prado ^2^, Laura González-Saiz ^4^, Elena Santana-Sosa ^2^, Natalia Yanguas-Casas ^2^, Eva SC-Ramos ^1^, Elena Higes-Núñez ^2^, Eva Garrido-Rodríguez ^2^, Joaquín Vico-Plaza ^2^, Carmen Fiuza-Luces ^2^ and Alejandro Lucía ^1^
^1^ Universidad Europea de Madrid, Faculty of Medicine, Health and Sports
^2^ Instituto de Investigación Sanitaria del Hospital Universitario 12 de Octubre (imas12)
^3^ Fundación Unoentrecienmil
^4^ Universidad Antonio de Nebrija

**Introduction:** Although exercise benefits in childhood cancer are documented, evidence focusing on adolescents is limited. This study aimed to evaluate the effects of an in-hospital physical exercise intervention on several health and fitness indicators in adolescents with cancer. **Methods:** A randomised controlled trial was conducted with 104 adolescents (12–19 years) newly diagnosed with (or having relapse of) malignant extracranial tumours. They were recruited from four public hospitals in Madrid (Spain). Participants were randomized (1:1) with a block on gender and tumour type (leukaemia/solid tumour). Controls received usual care plus counselling on healthy lifestyle, while the intervention group also performed supervised aerobic and resistance training thrice a week (mean ± SD adherence, 78 ± 20%). Training sessions were performed in the hospital gym, ward, or online. Outcomes were assessed at diagnosis and after post-intensive treatment. These included echocardiography-determined left ventricular function (primary outcome), muscle strength, functional mobility, dual-energy X-ray-absorptiometry-determined body composition, and clinical variables (survival, treatment tolerability, hospitalisation length). **Results:** No intervention-induced improvement was observed in left ventricular function. However, significant improvements were found in muscle strength (five-repetition-maximum pull-over test and maximal inspiratory pressure), functional capacity (30 s sit-to-stand test), and lean mass of the dominant upper limb (as assessed with dual-energy X-ray absorptiometry). Additionally, the intervention shortened hospitalisation length and reduced grade 2 and 4 adverse events, as well as the number of treatment delays. **Conclusions:** A supervised, individualized exercise program may enhance muscle strength, functional mobility, body composition, and clinical outcomes in adolescents undergoing intensive cancer treatment. Recruitment is ongoing, with a planned final sample of 136 participants and an additional follow-up at 3-month posttreatment.

### 7.4. Exercise During Pediatric Allo-HSCT: Follow-Up Results on Physical Function, Fatigue, and QoL from the Exploratory ANIMAL Trial

Ronja Beller ^1^, Gabriele Gauß ^1^, Oliver Basu ^1^, Stefan Schönberger ^1^, Michaela Höfs ^1^, Uta Dirksen ^1^ and Miriam Götte ^2^
^1^ Clinics for Pediatrics III, Department of Pediatric Hematology/Oncology, West German Cancer Centre, University Hospital Essen, Essen, Germany.
^2^ West German Cancer Centre, University Hospital Essen, Essen, Germany.

**Introduction:** Physical functioning, fatigue, and health-related quality of life (HRQL) are impaired during and after pediatric allogeneic hematopoietic stem cell transplantation (allo-HSCT). Exercise within the first 100 days may help support recovery. **Methods:** In the randomized controlled ANIMAL trial (DRKS00019865), 22 pediatric patients (≥4 years) undergoing allo-HSCT were allocated to a moderate–high- (IG^high^) or low-intensity (IG^low^) exercise group. Only IG^high^ continued with supervised sessions until day 100 (d100). Assessments of physical performance, fatigue, and HRQL were conducted at day 30 (d30, n = 21), d100 (n = 17), and one year post-transplant (d365, n = 14). Physical activity was measured using the ACTION questionnaire at d365. **Results:** At d100, IG^high^ showed higher leg strength than IG^low^ (n.s.) and greater parent-reported physical health (*p* = 0.043), with a trend toward lower fatigue (*p* = 0.054). At d365, IG^high^ performed better in sit-to-stand (*p* = 0.046), while IG^low^ showed higher right leg strength (*p* = 0.046). Change analyses revealed earlier improvements in IG^high^ (d30–d100) and later gains in IG^low^ (d100–d365). Both groups remained below age-specific reference values. At d365, only 30% of participants (IG^high^: 43%, IG^low^: 0%) met WHO physical activity guidelines. Participation in school/kindergarten sports was 60% overall, 20% engaged in club sports (exclusively IG^high^, non-competitive), while 50% reported non-club sports activities (IG^high^: 57%, IG^low^: 33%). Overall activity index (total: 30.9 MET hours/week) was markedly higher in IG^high^ (42.1 MET hours/week) compared to IG^low^ (4.9 MET hours/week). At d30, participation in physical performance tests and questionnaires was heterogeneous. By d100 and d365, participation differed between groups, with nearly complete assessments in IG^high^ compared to markedly lower rates in IG^low^, especially at d100. **Conclusions:** Moderate-intensity exercise during pediatric allo-HSCT appears to support earlier recovery of functional outcomes and higher long-term activity levels, underscoring the continued need for structured and supervised exercise programs in this population.

## 8. Research Poster Presentations

### 8.1. Adherence to Physical Activity Recommendations and Sedentary Time in Adult Survivors of Childhood Acute Lymphoblastic Leukemia—Associations with Fatigue and HRQL

Laura Jess, MSc, RPT ^1^, Katarina Aili, PhD, RPT ^2^, Jens M. Nygren, PhD ^3^, Maria Bäck, PhD, RPT ^4^ and Marianne Jarfelt, PhD, MD ^1^
^1^ Department of Oncology, Institute of Clinical Sciences, Sahlgrenska Academy, University of Gothenburg, Gothenburg, Sweden
^2^ Department of Health and Sport, School of Health and Welfare, Halmstad University, Sweden
^3^ School of Health and Welfare, Halmstad University, Sweden
^4^ Department of Molecular and Clinical Medicine, Institute of Medicine, Sahlgrenska Academy, University of Gothenburg, Gothenburg, Sweden

**Introduction:** The aim of this study was to examine whether the physical activity levels of adult survivors of childhood Acute Lymphocytic Leukemia (ALL) meet current physical activity recommendations, and to investigate how their physical activity and sedentary time are associated with fatigue and health-related quality of life (HRQL). **Methods:** This national cross-sectional study included 426 adult survivors of childhood ALL, diagnosed between 1985 and 2007 (49% women, median age 32, min-max 19–49), who responded to a questionnaire about physical activity (BHW-Q), sedentary time (SED-GIH-Q), fatigue (MFI-20), and HRQL (SF-36). Associations between physical activity, sedentary time, fatigue, and HRQL were analyzed using linear regression. **Results:** Overall, the physical activity levels of 44% of the participants did not meet current recommendations. Median weekly activity was 45 min (Q1–Q3 15–105) of vigorous and 105 min (Q1–Q3 45–175) of moderate-intensity activity, with a median sedentary time of 8 h (Q1–Q3 5–11) per day. Not meeting physical activity recommendations was significantly associated with increased fatigue (MFI-20 score, *p* = 0.01) and lower HRQL (MCS, *p* < 0.001 and PCS, *p* < 0.001). Sedentary time ≥ 7 h was also significantly associated with a lower mental health component of HRQL (MCS, *p* = 0.01). **Conclusions:** Many adult survivors of childhood ALL are insufficiently physically active and spend considerable time being sedentary. These results further emphasize the importance of promoting physical activity as a potential strategy to reduce fatigue and enhance HRQL in this population.

### 8.2. Childhood Cancer Survivors Exceed Healthcare Providers’ Expectations: The Power of Clinical Conversations

Maxime Caru ^1,2^, Lauryn E. Six ^3^, Brett Gordon ^2^, Smita Dandekar ^1^, Daniel J. McKeone ^1^, Pooja Rao ^1^, Christina Toepke ^1^, Michael Hayes ^4^, Nina S. Kadan-Lottick ^5^, Lisa M. McGregor ^1^ and Kathryn H. Schmitz ^6^
^1^ Department of Pediatrics, Division of Hematology and Oncology, Pennsylvania State Health Children’s Hospital, Hershey, PA, USA
^2^ Department of Public Health Sciences, Penn State University College of Medicine, Hershey, PA, USA
^3^ School of Kinesiology, Penn State’s College of Health & Human Development, Pennsylvania State University-Harrisburg, Middletown, PA, USA
^4^ Department of Psychiatry and Behavioral Health, Penn State University College of Medicine, Hershey, PA
^5^ Cancer Prevention and Control Program, Georgetown Lombardi Comprehensive Cancer Center, Washington, DC, USA
^6^ Division of Hematology and Oncology, University of Pittsburgh, Pittsburgh, PA, USA

**Introduction:** Healthcare providers (HCPs) can play an important role in promoting physical activity (PA) in children and adolescents diagnosed with cancer. Therefore, it is critical to identify key aspects of clinical conversations that lead to increased PA promotion to offer personalized care. We aimed (1) to document HCPs’ knowledge when recommending PA to childhood cancer survivors (CCSs), (2) to identify themes that emerged from clinical conversations promoting PA, and (3) to document changes in CCS’ self-reported PA. **Methods:** A total of 20 CCSs (8 females and 12 males; 14.1 ± 2.4 years old) participated in audio-recorded conversations. HCPs (3 females and 1 male) were medical oncologists and nurse practitioners. HCPs received a one-time, 20 min educational presentation about the benefits of PA. The conversations promoting PA were incorporated into the clinical visit. A thematic review of the data was conducted to identify emerging themes. At baseline, 1 week and 3 months, CCSs completed the original version of the Godin Leisure-Time Exercise Questionnaire to self-report their PA. **Results:** CCS mean age was 14.1 ± 2.4 years. HCPs had, on average, 6.8 years of professional experience in pediatric oncology. A total of six themes emerged from the clinical conversations: #1—Introduction of PA, #2—PA recommendations, #3—Goal setting, goal progression and goal assessment, #4—Finding solutions and opportunities to engage in PA, #5—Receptiveness, and #6—Benefits of PA. At baseline, 75% (N = 15) were identified as ‘active’, 5% (N = 1) as ‘moderately active’ and 20% (N = 4) as ‘insufficiently active/sedentary’. Following conversations, CCSs were more active, and their PA behavior was maintained at 3-month follow-up. **Conclusions:** HCPs can play a pivotal role in championing PA promotion in CCSs when they receive additional training. The themes that emerged from this study provide insightful information to refine PA promotion strategies that can be implemented into existing and future clinical PA programs. This work was funded by a grant from the Four Diamonds Research Funds.

### 8.3. Feasibility and Acceptance of a Combined Inpatient and Online Exercise Program for Children and Adolescents with Oncological Diseases During Medical Treatment

Lena Böhlke ^1^, Amrei Friedrich ^1^, Barbara Hero ^1^, Thorsten Simon ^1^, Nora Zoth ^2^ and Freerk T. Baumann ^2^
^1^ Department of Pediatric Hematology and Oncology, University Hospital Cologne, Kerpener Straße 62, 50,937 Cologne, Germany
^2^ Department I of Internal Medicine, Center for Integrated Oncology Cologne Bonn, University Hospital Cologne, Cologne, Germany

**Introduction:** Children and adolescents with cancer show a significantly reduced level of physical activity during their medical treatment. However, exercise therapy during oncological treatment has a positive effect on the physical and mental well-being of patients. One objective of the study is to determine the feasibility and acceptance of additional supervised, home-based, online exercise sessions for patients. **Methods:** The HAPPY study is a randomized, controlled trial with a 12-week intervention period, involving patients aged 6 to 17 years. Patients in the active control group will receive exercise therapy during their inpatient stay (number of sessions depends on the number of inpatient stays). The intervention group will also receive supervised online sessions during the outpatient phase (min. 24 sessions/12 weeks). Feasibility and adherence to the study protocol will be documented. In addition, the subjective motivation of the patients and their satisfaction with the exercise sessions will be recorded. **Results:** To date, the study has included 18 patients (14 male, 4 female). There have been no adverse events in either the active control group or the intervention group thus far. Online exercise sessions are currently considered feasible. Current data on the feasibility and acceptance of the intervention were presented at the congress. **Conclusions:** Supervised, home-based, online exercise sessions are feasible and safe to implement during the outpatient phases of medical therapy for children with cancer. Exercise sessions are well received by patients and can be integrated into their routines.

### 8.4. Redesigning Community-Based Exercise Programs for Adolescents Affected by Cancer: A Survey-Informed Approach

Carolina Chamorro Vina ^1,2^, Chantelle Ball ^1^, Amanda Wurz ^2,3^, Fiona Schulte ^4^ and S. Nicole Culos-Reed ^2,4^
^1^ Kids Cancer Care Foundation of Alberta
^2^ University of Calgary, Faculty of Kinesiology
^3^ University of Fraser Valley, School of Kinesiology
^4^ University of Calgary, Cumming School of Medicine

**Introduction:** Physical activity (PA) is recommended for adolescents diagnosed with cancer. The Pediatric Cancer Patients and Survivors Engaging in Exercise for Recovery (PEER) program is a community-based exercise program created at the University of Calgary and has been offered by Kids Cancer Care (KCC) since 2013. It provides structured PA for children with cancer; however, adolescents participate less frequently than younger children. Aim: To explore factors influencing adolescents’ PA participation to inform the development of an adolescent-specific PEER program. **Methods:** With informed consent, a survey was distributed to adolescents (12–17 years) diagnosed with cancer and adolescent-aged siblings at KCC summer camps (n = 90) and through the KCC membership (n = 160). The survey included demographic, medical, and PA questions (both closed and open-ended), including PA levels and perceived barriers and motivators. Closed-ended responses were analyzed descriptively; open-ended responses were analyzed using content analysis. **Results**: Sixty-two adolescents (Mage = 13 ± 1.89 years; 28 diagnosed with cancer, 34 siblings) completed the survey (n = 57 camp, n = 14 email). Most (76%) were not currently participating in PEER. Based on the Godin Leisure-Time Exercise Questionnaire, 89% of participants reported being physically active. The top barriers to PA included lack of time, energy, and motivation, while key motivators were health benefits, mood improvement, and enjoyment. Overall, most barriers and enablers overlapped between siblings and patients/survivors. Main themes emerged: physical, psychological, social, and opportunity-related factors. Preferred PA interests included badminton, volleyball, swimming, soccer, and climbing. **Conclusions:** Survey results guided the creation of a new adolescent-specific PEER program featuring five sport-based modules. The program is guided by the RE-AIM framework and addresses barriers, enablers, and preferences, incorporating social events to enhance engagement and support behaviour change. It provides support to manage factors like fatigue, confidence and social connection. Findings highlight the importance of integrating youth input in program design.

### 8.5. Navigating Ethical Challenges in the FORTEe Randomised Controlled Trial: A Multi-Centre Staff Survey on Exercise Intervention for Children and Adolescents Undergoing Cancer Treatment

Francesca Alt ^1,2^, Elias Dreismickenbecker ^1^, Francesca Lanfranconi ^3^, Adriana Balduzzi ^3,4^, Eila Watson ^5^, Hayley Marriott ^6^, Joachim Wiskemann ^7^, Nikolai Bauer ^7^, Rodolf Mongondry ^8^, Martin Kaj Fridh ^9^, Alejandro Lucia ^10^, Carmen Fiuza-Luces ^11^, Ronja Beller ^12^, Filippo Spreafico ^13^, Barbara Konda ^14^, Milica Stefanović ^15^, Heidi Diel ^1^, Mareike Kühn ^1^, Lena Wypyrsczyk ^1^, Norbert W. Paul ^2^, Marie A. Neu ^1,^* and Joerg Faber ^1,^*
^1^ University Medical Center of the Johannes Gutenberg-University Mainz, Childhood Cancer Center Mainz, Germany
^2^ University Medical Center of the Johannes Gutenberg-University Mainz, Institute for the History, Philosophy and Ethics of Medicine, Mainz, Germany
^3^ Fondazione Monza e Brianza per Il Bambino e La Sua Mamma, Centro Maria Letizia Verga, Monza, Italy
^4^ Fondazione IRCCS San Gerardo dei Tintori and School of Medicine and Surgery, Milano-Bicocca University, Monza, Italy
^5^ Oxford Brookes University, Faculty of Health Science, and Technology, Oxford Institute of Applied Health Research, Oxford, UK
^6^ Oxford Brookes University, Faculty of Health Science, and Technology, School of Sport, Nutrition and Allied Health Professions, Oxford, UK
^7^ Heidelberg University Hospital, Medical Faculty Heidelberg, Heidelberg University, National Center for Tumor Diseases Heidelberg, a partnership between DKFZ and Heidelberg University Hospital, Heidelberg, Germany
^8^ Centre de Lutte Contre le Cancer Léon Bérard, Lyon, France
^9^ University Hospital Copenhagen—Rigshospitalet, Copenhagen, Denmark
^10^ Universidad Europea de Madrid, Madrid, Spain
^11^ Research Institute of the Hospital, Madrid, Spain
^12^ University Hospital Essen, West German Cancer Center, Essen, Germany
^13^ Fondazione IRCCS Istituto Nazionale dei Tumori, Milan, Italy
^14^ Forma 3D Ltd., Ljubljana, Slovenia
^15^ University Medical Center Ljubljana, Ljubljana, Slovenia

**Introduction:** Exercise interventions are increasingly recognised as valuable components of supportive care in paediatric oncology. However, their implementation within clinical trials raises ethical and psychosocial challenges. The FORTEe randomised controlled trial (NCT05289739) evaluates the effects of a structured exercise programme for children and adolescents with cancer. This survey aimed to capture healthcare professionals’ perspectives (HCPs) on ethically challenging aspects of implementing the trial. **Methods:** A multicentre survey was conducted at ten European FORTEe sites, where HCPs completed a structured questionnaire containing quantitative and qualitative components, which were analysed descriptively and thematically. Explored domains included informed consent and assent, parental influence, the assessment of burden and benefit, allocation to the control group, the sustainability of interventions, and moral distress. **Results:** Seventy-nine HCPs participated, including exercise professionals, physicians, nurses, psychologists and social workers. Of the HCPs, 80% judged benefits to outweigh burdens, reporting improvements in patients’ recovery and well-being. Challenges included interpreting children’s assent, parental influence on decision-making and burdens from questionnaires and logistics. Control group allocation caused disappointment for some families and emotional discomfort for staff. Nearly 69% of HCPs reported moral distress, particularly related to relapse, disease progression, or death. While some centres successfully integrated exercise programmes into standard care, others noted sustainability gaps and inequities in access beyond the trial. **Conclusions:** While the trial was generally perceived as ethically sound, tensions remained. Priorities include child-centred communication, safeguarding fairness, post-trial access, and staff support in managing moral distress. The FORTEe experience demonstrates how ethically responsive frameworks can promote equitable and child-centred integration of exercise into paediatric cancer care. These insights provide guidance for future paediatric exercise trials, highlighting the need for designs addressing both patient and staff perspectives.

Funding: This project has received funding from the European Union’s Horizon 2020 research and innovation programme, under grant agreement No 945153.

### 8.6. The PED-SARC-F as a Screening Tool for Physical Function After Pediatric Hematopoietic Stem Cell Transplantation

Marloes W. B. Visser ^1^, Emma den Hartog ^1^, Wim J. E. Tissing ^1,2^ and Emma J. Verwaaijen ^1^
^1^ Princess Máxima Center for Pediatric Oncology, Utrecht, The Netherlands
^2^ Department of Pediatric Oncology and Hematology, University of Groningen, Beatrix Children’s Hospital, University Medical Center Groningen, Groningen, The Netherlands

**Introduction:** Allogeneic Hematopoietic Stem Cell Transplantation (HSCT) is an intensive procedure for children with hematological malignancies and non-malignant disorders, often resulting in long-term impairments in physical function. Early identification of children at risk of reduced physical function is crucial to optimize care and prevent long-term limitations. However, physical performance tests are not always feasible. The Pediatric SARC-F (PED-SARC-F) is a non-physical screening tool for assessing physical function in pediatric oncology patients. This study examined whether the PED-SARC-F, administered prior to HSCT (pre-HSCT), is associated with physical function approximately 100 days after HSCT. **Methods:** In this retrospective cohort study, children aged 3–18 years who underwent allogeneic HSCT at the Princess Máxima Center for Pediatric Oncology, the Netherlands, between 2018 and 2023 were eligible. As part of standard care, physiotherapy consultations were performed pre- and post-HSCT. Pre-HSCT PED-SARC-F scores and post-HSCT physical function assessments were extracted from patient records. Physical function was evaluated using the Time to Rise from the Floor (TRF), Ten Meter Walk Test (10-MWT), Ten Meter Run Test (10-MRT), and Timed Up and Down Stairs (TUDS). Multivariable linear regression models were used to study associations between pre-HSCT PED-SARC-F scores and post-HSCT physical function. **Results:** A total of 49 patients were included (mean age 11.7 ± 3.7 years; 71% male). Higher pre-HSCT PED-SARC-F scores were significantly associated with poorer post-HSCT performance on the TUDS (β = 1.715, 95%CI = 0.790–2.640) and 10-MRT (β = 0.125, 95%CI = 0.007–0.242), in which higher test scores mean slower performance. PED-SARC-F scores were not associated with TRF and 10-MWT post-HSCT. **Conclusions:** Pre-HSCT PED-SARC-F scores were associated with impaired stair climbing and running performance 100 days after HSCT, but not with rising from the floor or walking speed. The PED-SARC-F may serve as a simple and feasible screening tool to identify children at risk of specific post-HSCT physical function impairments.

### 8.7. How the FORTEe Project Impacted Precision-Based Exercise Perception at Istituto Nazionale Tumori

Wiliam Zardo ^1,2,3^, Diletta Gazzaroli ^3^, Marco Chisari ^1^, Michele Murelli ^1^, Gloria Tarantino ^1^, Sabrina Zucchi ^1^, Paolo Grampa ^1^, Maura Massimino ^1^, Eliana Roveda ^2^, Caterina Gozzoli ^3^ and Filippo Spreafico ^1^
^1^ Paediatric Oncology Unit, Fondazione IRCCS Istituto Nazionale dei Tumori, Milan, Italy
^2^ Department of Biomedical Sciences for Health, University of Milan, Italy
^3^ Department of Psychology, Università Cattolica del Sacro Cuore, Milan, Italy

**Introduction:** Survival rates for children, adolescents, and young adults with cancer (CAYAs) have significantly improved due to advances in care and personalized treatments. However, side effects of therapies often limit patients’ ability to engage in sports and exercise during treatment. These effects can be reduced or mitigated through precision-based exercise (PEx). Despite growing evidence supporting the benefits of PEx, it has yet to be routinely implemented in all pediatric oncology units. In 2020, the European project FORTEe was launched to implement evidence for a more consistent and comprehensive integration of PEx into clinical routine. Istituto Nazionale dei Tumori (INT), among the clinical partners, already had a Sport Project since 2013. This study aimed to explore how the FORTEe project influenced the integration and delivery of PEx at INT. **Methods:** Fifteen professionals—including pediatricians/oncologists, exercise physiologists, psychologists, physiotherapists, and educators—participated in semi-structured interviews. Four thematic areas were explored: concept of care; care and sport; sport project in hospital and reproducibility of the project. **Results:** Participants widely recognized and valued the existing Sport Project and reported positive changes after the start of FORTEe—particularly the presence of a dedicated exercise physiologist and a more structured “exercise” routine. Increased engagement from healthcare personnel in identifying and referring eligible patients was noted, although not all pediatricians/oncologists remained proactive in proposing PEx after enrolment. Effective collaboration between exercise professionals and healthcare staff was deemed essential for daily adaptation of exercise sessions to each child’s clinical status. **Conclusions:** The FORTEe study significantly strengthened the implementation of PEx within clinical practice at INT. Notably, it reinforced the key importance of interdisciplinary collaboration, fostering daily communication between exercise professionals and clinical staff to ensure individualized exercise sessions for CAYAs.

### 8.8. Enriching the Ward Environment via an Activity Coordinator to Improve Physical and Daily Activity Levels in Paediatric Oncology

Vicky King ^1^ and Rebecca Tapley
^1^ Brainbow, Neuro-Oncology Rehabiliation, Cambridge University, Cambridge, United Kingdom

**Introduction:** Data collected during a Patient Experience Project at Cambridge University Hospital showed that when children entered oncology wards, they showed a decline in both physical (e.g., exercise and active play) and daily activity levels (e.g., washing and dressing) compared to when at home, even when medically able to participate. **Methods and Results:** The qualitative feedback gathered from patients, families and staff indicated that it was mainly environmental and cultural factors within the ward that prevented activity and movement, such as a lack of opportunity, a lack of role modelling and a culture that focused on sickness/inability over activity. Easier options are often sought and a “sick role” is established, with limited expectation, encouragement, promotion or permission to engage in activity, and when activity was sought by the child, there was neither the opportunity nor the accessibility to enable it. These environmental reasons were cited more frequently than patient or physiological issues such as medications, infection rules, weakness, fatigue or fear; however, most therapeutic interventions to improve activity currently focus on the individual rather than the wider ward. **Conclusions:** We therefore decided to target both physical and daily activity levels at a universal ward level by employing an Activity Coordinator dedicated to encouraging active movement (both physical and daily) throughout the ward through education, modelling and provision of additional activity alongside our nursing, play and education colleagues. We will present our preliminary findings, feedback and reflections from this endeavour.

### 8.9. Building Capacity and Advancing Exercise Oncology Practice: Developing a Pediatric Exercise Oncology Training Program for Fitness Professionals

Amanda Wurz ^1^, Emma McLaughlin ^2^, Lauren Ha ^3^ and S. Nicole Culos-Reed ^2^
^1^ Kinesiology, University of the Fraser Valley, 30-5960 Cowichan St, V2R0L6 Chilliwack, Canada
^2^ Faculty of Kinesiology, University of Calgary, Calgary, Canada
^3^ School of Paediatrics and Child Health, School of Clinical Medicine, University of New South Wales, Australia

**Introduction:** Physical activity (PA), particularly supervised PA, is safe and beneficial in pediatric oncology. However, limited training opportunities exist to equip fitness professionals with the knowledge necessary to deliver safe and engaging PA to children and adolescents with cancer. This lack of training poses a barrier to widespread PA implementation. We are developing a module-based, self-directed, online pediatric exercise oncology training program to provide fitness professionals the knowledge needed to deliver safe and engaging PA to this cohort. **Methods:** An outline was iteratively drafted based on the available literature, the authorship team’s substantive knowledge and expertise in pediatric exercise oncology, and other available oncology-specific training (i.e., the third author’s adult exercise oncology training). The outline was then sent to nine international experts who critically reviewed and approved it and shared content to be included within the modules. Modules are currently being drafted, reviewed, and refined, and literature updates are being performed to ensure the inclusion of the most up-to-date evidence. It is anticipated that the initial draft will be completed in December 2025. Following this, module content will undergo critical review by >12 international experts to ensure the training is comprehensive and accurate. The training is anticipated to be launched in May 2026. **Results:** Training modules will cover the following topics: pediatric cancer and treatments, physical and psychosocial impacts of cancer, evidence and principles of PA, common considerations and contraindications, implementing PA interventions, and PA behavior change support. **Conclusions:** This work represents a critical step toward competency building in pediatric exercise oncology and will help ensure a broader range of fitness professionals (e.g., personal trainers, group fitness instructors, kinesiologists) gain the knowledge necessary to deliver safe and engaging PA. Feedback, in the form of open- and close-ended questions, from those accessing the training will be collected to assess utility, effectiveness, satisfaction, and areas for improvement.

### 8.10. Moving Forward Together: Co-Adapting and Preparing to Scale an Online Physical Activity Intervention for Pediatric Cancer and Blood Disorder Patients in Canada

Lauren Ha ^1,2^, Vanessa Sales ^1^, S. Nicole Culos-Reed ^3^, Caron Strahlendorf ^4^, Kristin Marr ^5^, Hanna Lotocka-Reysner ^6^, Ewa Lunaczek-Motyka ^7^, Anne Carrelli ^8^, Stuart Peacock ^9^, Iris Lesser ^1^, Sharon Hou ^10^, Fuchsia Howard ^11^, Kristin Campbell ^12^, Brianna Empringham ^13^, Raveena Ramphal ^13^, Lillian Sung ^14^, Donald Mabbott ^14^, Chelsea Ash ^15^, Anette Flanders ^15^, Mary Stuart ^15^, Melanie Keats ^16^, Eden Baerg ^1^, Emma McLaughlin ^3^, Megan Filiatrault ^1^ and Amanda Wurz ^1^
^1^ School of Kinesiology, University of the Fraser Valley, Chilliwack, BC, Canada
^2^ School of Clinical Medicine, University of New South Wales, Sydney, Australia
^3^ Faculty of Kinesiology, University Calgary, Canada
^4^ Division Pediatric Hematology/Oncology/BMT, British Columbia Children’s Hospital, Vancouver, BC, Canada
^5^ Pediatric Oncology Clinic, Surrey Memorial Hospital, Canada
^6^ Pediatric Oncology Clinic, Surrey Memorial Hospital, Canada
^7^ Victoria General Hospital—Pediatric Oncology Program
^8^ Victoria General Hospital—Pediatric Oncology Program, Canada
^9^ Faculty of Health Sciences, Simon Fraser University, Canada
^10^ Faculty of Education, Simon Fraser University, Canada
^11^ School of Nursing, University of British Columbia, Canada
^12^ Department of Physical Therapy, University of British Columbia, Canada
^13^ Children’s Hospital of Eastern Ontario, Canada
^14^ The Hospital for Sick Children, Canada
^15^ Division of Hematology-Oncology, IWK Health, Canada
^16^ School of Health and Human Performance, Dalhousie University, Halifax, NS, Canada

**Introduction:** Physical activity (PA) is safe and beneficial for pediatric cancer and blood disorder patients, yet PA levels are low. We developed a videoconference-delivered PA intervention called ‘IMPACT’ (IMplementation of Physical Activity for Children and adolescents on Treatment). We are evaluating IMPACT in Alberta and will expand to additional sites in Canada (British Columbia [BC], Ontario [ON], the Maritimes). Given regional diversity, the IMPACT intervention requires adaptation to ensure relevance, acceptability, and sustainability. Aims: To co-adapt IMPACT for implementation and evaluation and generate evidence that informs a model for ongoing adaptation and scaling. Specific objectives include: (i) identifying necessary IMPACT modifications and (ii) examining factors influencing IMPACT implementation. **Methods:** A multi-perspective study adopting a patient-oriented research approach is underway. Descriptive surveys and 1:1 interviews, guided by the Consolidated Framework for Implementation Research 2.0, are being conducted with pediatric cancer and blood disorder patients/survivors and their carers, healthcare professionals, and support organization representatives. Data are being analyzed using descriptive statistics and thematic and framework analyses. **Results:** As of September 2025, one survivor (13-year-old male diagnosed with leukemia) and their carer, six healthcare professionals (2 = oncologist, 2 = physiotherapist, 2 = nurse clinicians), and one support organization representative have been interviewed. Additional interviews at all sites are planned. Early findings suggest modifications to delivery style (i.e., hybrid approaches) and participant population (i.e., expanding to include patients during and after treatment). Factors that may influence IMPACT implementation include healthcare professional referral capacity and patient/survivor symptom burden and motivation for PA. **Conclusions:** Adapting IMPACT with patients, families and healthcare professionals will enhance acceptability, relevance, and sustainability. Results will directly inform the co-development of an implementation research logic model and will provide a firm foundation upon which a trial evaluating site-specific IMPACT can be conducted. This work reimagines IMPACT for broader applicability across diverse Canadian contexts.

### 8.11. Effectiveness of Home-Based Exercise Programs in Children with Acute Lymphoblastic Leukemia: A Systematic Review

Sena Bılazer ^1^, Vesile Yıldız Kabak ^2^ and Songul Atasavun Uysal ^2^
^1^ Department of Occupational Therapy, Health Services Vocational School, Eurasia University, Trabzon Turkiye.
^2^ Department of Physiotherapy and Rehabilitation, Faculty of Health Sciences, Hacettepe University, Ankara, Turkiye.

**Introduction:** The purpose of this review is to synthesize the existing scientific literature on physical fitness levels and home-based exercise interventions in children with acute lymphoblastic leukemia (ALL). **Methods:** The review process was guided by the Preferred Reporting Items for Systematic Reviews and Meta-Analyses checklist. A systematic search was performed in the PubMed and Web of Science databases, with the final search conducted on 17 December 2024. The search terms were (home-based OR home based OR home OR home-setting OR home care) AND (physical therapy OR intervention OR rehabilitation OR exercise OR physical activity OR training OR walking OR activity) AND (Child cancer OR leukemia OR lymphoma OR bone tumor OR brain tumor OR pediatric cancer OR hematopoietic stem cell transplantation OR bone marrow transplantation OR childhood cancer survivor). **Results:** Based on the initial search terms, a total of 946 articles were identified through the database search, and 1 additional eligible study was found by reviewing the reference lists. After removing duplicates, 653 articles remained, and their titles and abstracts were screened. Of these, 638 articles were excluded for not meeting the eligibility criteria. The full texts of the remaining 15 articles were then assessed against the inclusion criteria. Following this full-text review, five articles that met the inclusion criteria were included in the qualitative synthesis. **Conclusions:** Home-based exercise interventions have the potential to improve physical health parameters in children with ALL. However, further research in this field should be conducted with larger and more homogeneous sample sizes, adhering to stricter methodological standards.

### 8.12. The Use of an Augmented Reality App to Support an Exercise Intervention for Children and Young People with Cancer: Perspectives of Users and Healthcare Professionals in the FORTEe Trial

Hayley Marriott ^1,^*, Alba Solera-Sanchez ^1,^*, Stanley Windsor ^1^, Kim Straun ^3^, Marie Neu ^4^, Elias Dreismickenbecker ^4^, Francesca Lanfranconi ^5^, Emanuele Villa ^5^, Joachim Wiskemann ^6^, Nikolai Bauer ^6^, Miriam Götte ^7^, Ronja Beller ^8^, Filippo Spreafico ^9^, William Zardo ^9^, Peter Wright ^1^, Joerg Faber ^4,^** and Eila Watson ^2,^** on behalf of the FORTEe Consortium
^1^ School of Sport, Nutrition and Allied Health Professions, Faculty of Health Science, and Technology Oxford Brookes University, Oxford, UK
^2^ Oxford Institute of Applied Health Research, Faculty of Health Science, and Technology, Oxford Brookes University, Oxford, UK
^3^ School for Policy Studies, University of Bristol, UK
^4^ University Medical Center of the Johannes Gutenberg-University Mainz, Childhood Cancer Center Mainz, Mainz, Germany
^5^ Fondazione Monza e Brianza per Il Bambino e La Sua Mamma, Monza, Italy
^6^ Working Group Exercise Oncology, Department of Medical Oncology, Heidelberg University Hospital, Medical Faculty Heidelberg, Heidelberg University, National Center for Tumor Diseases Heidelberg, a partnership between DKFZ and Heidelberg University Hospital, Heidelberg, Germany
^7^ University Hospital Essen, West German Cancer Center, Essen, Germany
^8^ Clinics for Paediatrics III, Department of Paediatric Haematology/Oncology, West German Cancer Centre, University Hospital Essen, Essen, Germany.
^9^ Fondazione IRCCS Istituto Nazionale dei Tumori, Pediatric Oncology Unit, Milan, Italy
* these authors share first authorship
** these authors share last authorship

**Introduction:** Mobile health (mHealth) technologies are increasingly used in paediatric oncology to promote physical activity, with growing evidence of feasibility and effectiveness. Augmented reality (AR) offers interactive and engaging features that may further enhance participation, especially when in-person provision is limited. As mHealth tools evolve, understanding user experiences and implementation challenges is essential for wider adoption. This study explored the perspectives of children, adolescents and young adults with cancer (CAYAs) and exercise and healthcare professionals (EHCPs) on a novel AR application (app) within the FORTEe clinical trial. **Methods:** CAYAs (aged 9–21 years) and EHCPs from the FORTEe technology sub-study (six centres across Europe) were eligible. The app was designed to facilitate home-based exercise as part of a broader individualised exercise programme. App features included personalised exercise programmes, AR demonstrations by a child-friendly avatar, and an integrated exercise diary. Half-structured interviews were conducted with CAYAs, and an anonymous online survey was administered to EHCPs. **Results:** In total, 46 CAYAs provided feedback and 31 EHCPs completed the online survey. Both groups reported positive experiences overall, highlighting the novelty and engagement of AR demonstrations and the value of personalised workouts. However, technical difficulties occasionally affected the reliability of AR functions, and features such as the diary were considered less user-friendly. EHCPs reported that the app should complement rather than replace face-to-face sessions. Suggestions for improvement included gamification and avatar customisation. **Conclusions:** AR technology shows promise in enhancing engagement with exercise among CAYAs with cancer. This study highlights the importance of user-friendly design, personalisation, and reliable functionality, alongside the need to maintain in-person support. Future development should prioritise co-development, optimising usability, and ensuring equitable access for CAYAs and their families.

### 8.13. Prevalence of Pain and Its Association with Tumor Location and Physical Activity in Pediatric Brain Tumor Survivors: A Master’s Thesis

Hanna Lundqvist ^1^, Åsa Persson Dobruna ^1^ and Sara Frygner-Holm ^1^
^1^ Uppsala Universitet

**Introduction:** Children treated for brain tumors often experience long-term health challenges. Pain is a known late effect in pediatric oncology, but its prevalence and impact among brain tumor survivors are not well understood. This study aims to investigate the prevalence of pain in children who have completed treatment for brain tumors, and to explore associations between pain, tumor location, physical activity, and sedentary behavior. **Methods:** This cross-sectional study includes children who completed brain tumor treatment at least six months prior. Data are collected via questionnaires assessing pain, physical activity, and sedentary behavior. Tumor location and clinical background were retrieved from the Swedish National Childhood Cancer Registry. Statistical analyses will explore differences in pain prevalence by tumor location and associations with activity levels. **Results:** To date, 113 children (56 boys, 57 girls) and 123 legal guardians have responded. Data collection will conclude in October 2025. Preliminary findings indicate that pain is reported by X% of participants. Final results will describe differences in pain prevalence by tumor location and compare physical activity and sedentary behavior between children with and without pain. **Conclusions:** This study is expected to provide new insights into pain and its relationship with physical activity in pediatric brain tumor survivors, contributing to improved follow-up care and rehabilitation strategies.

### 8.14. Adapted Exercise and School in Hospital: The PLAYFIELD Framework Promotes an Alliance to Improve Learning in Children, Adolescents and Young Adults with Haematological Malignancies

Marta Corti ^1^, Tommaso Moriggi ^1^, Linda Peli ^1^, Eleonora Corti ^1^, Emanuele Villa ^1^, Simona Ferrari ^2^, Paolo Raviolo ^3^, Orianna Marzi ^4^, Maria R. Maggioni ^4^, Federica Lavagnini ^4^, Lorena Almansi ^5^, Momcilo Jankovic ^1^, Adriana C. Balduzzi ^6,7^ and Francesca Lanfranconi ^1^
^1^ Fondazione Monza e Brianza per il Bambino e la sua Mamma—Centro Maria Letizia Verga, Monza (Italy)
^2^ Università Cattolica del Sacro Cuore Milano—CREMIT
^3^ Università E-Campus
^4^ Istituto Comprensivo Salvo D’Acquisto
^5^ Istituto Statale di Istruzione Superiore Mosè Bianchi
^6^ Università degli Studi di Milano Bicocca, School of Medicine and Surgery, Milan (Italy)
^7^ Fondazione IRCCS San Gerardo dei Tintori, Monza (Italy)

**Introduction:** Exercise has beneficial effects on cognition, academic performance and skill development. School-in-hospital teachers (SHTs) support children and adolescents in cancer treatment (CA-h) through a personalised relationship. SHTs are not allowed to use exercise to improve academic performance in hospital. Precision-based exercise programmes (PEx) are a new option to improve exercise tolerance and quality of life in CA-h. New alliances between exercise professionals and pedagogues can be beneficial within the academic curricula of any CA-h. The PLAYFIELD framework provides the pillars to build this integrated multidisciplinary knowledge. **Methods:** The study tested educational activities developed by teachers from Lombardy’s SIOs and teachers from the Collegio Villoresi in Monza, following voluntary training based on the PLAYFIELD framework. The training, conducted by a multidisciplinary team of sports doctors, educators and motor scientists, involved their essential contribution in identifying and validating safe and adapted physical exercises. The team worked closely with the teachers, supporting them in integrating the exercises into their teaching activities and promoting motor skills as part of the educational process. **Results:** Forty-one teaching cards were produced, including EF exercises adapted to different school levels: four for nursery school, 17 for primary school, 10 for lower secondary school and 10 for upper secondary school. **Conclusions:** SHTs integrated PEx into the classroom and improved students’ academic performance and developmental skills. Future research will explore the impact of the PLAYFIELD methodology through a randomised trial, assess satisfaction through questionnaires, and explore opportunities for interdisciplinary collaboration between primary and secondary schools to promote continuity and adaptation of the methodology.

### 8.15. Physiological Effects of Exergaming on Muscle Strength, Balance and Cancer-Related Fatigue in Children with Cancer

Ruttvi Dagudu ^1^, Damanbir Singh Sandhu ^2^, Cynric Lobo ^3^ and Nivedita Prabhu ^4^
^1^ Department of Physiotherapy, Manipal College of Health Professions, Manipal Academy of Higher Education, #8 Sunbeam, Deonar Baug, Govandi(east), Mumbai-400088, Maharashtra, India
^2^ Physiotherapy, Manipal College of Health Professions, Manipal Academy of Higher Education, House no 1073, Phase 10, Mohali, Inner Rd, Sahibzada Ajit Singh Nagar, Sector 64, India
^3^ Physiotherapy, Manipal College of Health Professions, Manipal Academy of Higher Education, Raje Shivaji nagar, 400059 Lok-Darshan Society, Marol Maroshi road, Andheri East, Mumbai, India
^4^ Physiotherapy, Manipal College of Health Professions, Manipal Academy of Higher Education, 576104 Manipal, Karnataka, India

**Introduction**: Children with cancer and survivors thereof experience chronic physical problems such as reduced muscle strength, balance difficulties, and persistent cancer-related fatigue. These impairments may dually arise from both the cancer and treatment regimens, leading to muscle atrophy, impaired neuromuscular control and general deconditioning. Conventional exercise regimens are effective, yet they can be both monotonous and tiring. Exergaming combines exercise with interactive video games, providing an engaging and enjoyable alternative. We aimed to explore the physiological changes that occur through exergaming and how these may improve muscle strength, balance, and fatigue in this population. **Methods**: Exergaming enhances muscle strength by engaging larger muscle groups (lower limbs and trunk) through repetitive, closed kinematic chain movements, subsequently stimulating motor unit recruitment, developing improved coordination and promoting hypertrophy. Restoration of protein synthesis, improved blood flow, and increased mitochondrial function together contribute to improved muscle strength and endurance. **Results:** Concomitant adjustments in postural control during exergaming and rapid visual-auditory cue reactions challenge the vestibular system, proprioception and visual processing. Repeated practice promotes sensory integration and refines both anticipatory and reactive postural control. These adaptations are particularly important for children whose balance is affected by peripheral neuropathy, as they lead to steadier gait, reduced risk of falls, and greater independence in everyday activities. Physiologically, moderate-intensity activity improves cardiovascular conditioning, enhances mitochondrial efficiency and reduces systemic inflammation. Psychologically, the interactive and playful nature of exergames provides motivation and excitement, lowering the perception of fatigue. This combination supports better energy regulation, a positive mood and improved participation in daily routines. **Conclusions**: Exergaming presents a promising, child-friendly approach in counteracting the physical effects of cancer and its treatment by promoting neuromuscular activation, balance retraining, cardiovascular fitness, and psychological engagement.

### 8.16. The 6 min Cycling Test to Assess Endurance Capacity in Childhood Cancer Patients

Lena Wypyrsczyk ^1^, Mareike Kühn ^1^, Elias Dreismickenbecker ^1^, Marie A. Neu ^1^ and Joerg Faber ^1^
^1^ Pediatric Hematology/Oncology, Department of Pediatrics, University Medical Center of the Johannes Gutenberg-University Mainz, Mainz, Germany.

**Introduction:** The 6 min cycling test (6MCT) is a submaximal endurance test that has yet to be employed in pediatric oncology. The aim of this study was to investigate the feasibility and validity of the 6MCT in childhood cancer patients. **Methods:** A total of 71 children, adolescents and young adults diagnosed with cancer (mean age 9.6 ± 4.0 years, 53.5% male) were recruited to perform the 6MCT on a cycle ergometer. To validate the test, 46 patients additionally underwent cardiopulmonary exercise testing (CPET), which is considered the gold standard for assessing cardiorespiratory fitness. The correlation between peak oxygen uptake (VO2peak) and peak work rate (WRpeak) measured during CPET and the results of the 6MCT was determined using the Spearman correlation coefficient (ρ). Two simple linear regression models were used to predict the impact of VO2peak and WRpeak on 6MCT performance. **Results:** A total of 66 patients (93%) successfully completed the 6MCT. The average revolutions achieved in six minutes was 550 ± 129, with a mean heart rate of 157 ± 25 beats per minute. There was a positive moderate correlation between revolutions at the end of the 6MCT and VO2peak (ρ = 0.46, *p* = 0.001), and a positive large correlation with WRpeak (ρ = 0.64, *p* < 0.001). Linear regression demonstrated that VO2peak was a significant predictor of performance during the 6MCT (b = 5.371; *p* = 0.001, R^2^ = 0.214), while WRpeak explained more variance (b = 98.465; *p* < 0.001, R^2^ = 0.488). **Conclusions:** The results demonstrate that the 6MCT is a feasible and valid method for measuring endurance capacity in childhood cancer patients, offering a promising alternative when gold standard testing is not available. This project has received funding from the European Union’s Horizon 2020 research and innovation programme, under grant agreement No 945153.

### 8.17. Cross-Centre Analysis of Childhood Cancer Patients’ Experiences Within the FORTEe Exercise Trial: Insights from Denmark and Germany

Francesca Alt ^1,2,^*, Marie A. Neu ^1,^*, Heidi Diel ^1^, Hanne Bækgaard Larsen ^3^, Martin Bjørn Hansen ^3^, Elias Dreismickenbecker ^1^, Dennis Wilke ^2^, Norbert W. Paul ^2^, Martin K. Fridh ^3,‡^ and Joerg Faber ^1,‡^
^1^ University Medical Center of the Johannes Gutenberg-University Mainz, Childhood Cancer Center Mainz, 55131 Mainz, Germany
^2^ University Medical Center of the Johannes Gutenberg-University Mainz, Institute for the History, Philosophy and Ethics of Medicine, 55131 Mainz, Germany
^3^ University Hospital Copenhagen—Rigshospitalet, Department of Pediatrics and Adolescent Medicine, 2100 Copenhagen, Denmark
* FA and MAN share first authorship
^‡^ MKF and JF share last authorship

**Introduction:** Exercise therapy is increasingly recognised as a supportive treatment in paediatric oncology, helping to mitigate loss of physical fitness and preserve quality of life. While single-centre studies show clear benefits of exercise interventions, little is known about how cultural and healthcare contexts shape families’ experiences. This study compared patient and parent perspectives from the German and Danish sites of the FORTEe trial. Uniquely, the Danish cohort included both intervention and control group participants. **Methods:** Semi-structured interviews were conducted with 23 participants: 9 from Mainz, Germany (6 patients, 3 parents; intervention only) and 14 from Copenhagen, Denmark (7 patients, 7 parents; intervention and control). Data were transcribed, translated where necessary, and analysed using reflexive thematic analysis and the Framework Method to explore convergent and divergent themes across countries and treatment groups. **Results:** Across both sites, families described enhanced physical capability, psychological distance from illness and recognition as individuals. In Denmark, three additional themes were generated: (1) strategies to stay physically active (caregiver support, gamification and daily routines); (2) family health beliefs (exercise as medicine, normalcy restoration and social cohesion); and (3) experiences of being randomized into the control group. The latter ranged from disappointment and perceived exclusion to acceptance, relief from the training burden and a positive identification with the research project. **Conclusions:** Exercise therapy was valued across both countries for its physical and psychological benefits, while Danish data revealed additional insights into activity strategies, family health beliefs, and control group experiences. These findings show that family dynamics and cultural context influence how exercise therapy is received, highlighting the need for context-sensitive and equitable integration into paediatric oncology care.

Funding: This project has received funding from the European Union’s Horizon 2020 research and innovation programme, under grant agreement No 945153.

### 8.18. Home-Based Intervention with Physical Activity Booklets: Impact on Cancer-Related Fatigue in Pediatric Patients

Cintia de la Rocha Freitas ^1^, Micheli Carminatti ^2^, Isadora Dalla Lana ^2^, Érica Cappellaro ^2^, Ingrid Alessandra Victoria Wolin ^2^ and Licelli Amante Cardoso ^3^
^1^ Physical Education Postgraduate Program, Federal University of Santa Catarina, Campus Universitário Trindade, s/n, 88035-972 Florianopolis, Brazil
^2^ Physical Education Postgraduate Program, Prédio Administrativo Centro de Desportos, Federal University of Santa Catarina, Campus Universitário Trindade, s/n, 88035-972 Florianopolis, Brazil
^3^ Human Movement Sciences Postgraduate Program, Santa Catarina State University, Brazil

**Introduction:** Childhood cancer, in addition to compromising survival, produces significant adverse effects during treatment, among which cancer-related fatigue is one of the most disabling symptoms. This study aimed to analyze the effects of a home-based physical activity program implemented through Physical Activity Booklets. **Methods:** This was an uncontrolled clinical trial conducted with 25 participants (aged 4–14 years) undergoing outpatient treatment. The booklets were specifically designed for two age groups (4–11 years; 12–14 years) and contained playful or functional activities to be performed twice a week for 12 weeks. Fatigue was monitored using pictograms and perceived exertion scales. Fatigue outcomes were assessed using the Pediatric Quality of Life Inventory—Multidimensional Fatigue Scale, in both patient and caregiver versions. Analyses were conducted by intention-to-treat (ITT) and per-protocol (PP). **Results:** Mean adherence to the booklets was 77.6% ± 17.8%. In the ITT analysis, a significant improvement in total fatigue was observed as reported by both patients (*p* = 0.039) and caregivers (*p* = 0.014), with notable improvements in the sleep/rest (*p* < 0.001) and general fatigue (*p* = 0.012) domains. In the PP analysis, beyond total fatigue (patients *p* = 0.038; caregivers *p* = 0.012), positive effects were also found for the sleep/rest (*p* = 0.023) and general fatigue (*p* = 0.009) domains. Weekly pictogram monitoring indicated a progressive reduction in symptoms, from “very/extremely tired” to “a little tired” by the 12th week. **Conclusions:** The intervention proved effective in reducing cancer-related fatigue, leading to clinically meaningful improvements in overall fatigue perception, particularly in physical and sleep-related aspects. This model represents a feasible, low-cost, home-based strategy with potential as a complementary support to pediatric cancer treatment.

### 8.19. A Physical Activity Program to Improve Cardiorespiratory Fitness in Children and Adolescents Following Acute Cancer Treatment (POWER): Current Status of a Randomized Controlled Trial

Sandra Goertz ^1^, Benedikt Bötticher ^1^, Tim Niehues ^2^, Wolfgang Lawrenz ^3^, Gabriele Gauß ^4^, Corina Silvia Rueegg ^5^ and Miriam Götte ^4^
^1^ Pediatric Oncology, Helios Hospital Krefeld, Am Buschkamp 9a, 47239 Duisburg, Germany
^2^ Center for Pediatric and Adolescent Medicine, Helios Hospital Krefeld, Germany
^3^ Pediatric Cardiology, Helios Hospital Krefeld, Germany
^4^ Clinics for Pediatrics III, Department of Pediatric Hematology/Oncology, West German Cancer Centre, University Hospital Essen, Essen, Germany
^5^ Department of Biostatics, University of Oslo, Norway

**Introduction:** Treatment of pediatric cancer leads to reduced physical activity and performance. Structured exercise may counteract and mitigate treatment side effects and support re-integration after treatment cessation. The primary aim of the POWER-RCT is to evaluate the effectiveness of a partially supervised 12-week physical activity program on cardiorespiratory fitness (VO2 peak) in pediatric cancer patients who have recently completed acute treatment. Secondary outcomes are muscular strength, functional mobility, coordination, cognitive function, inflammatory and cardiac parameters, physical activity levels, health-related quality of life, and fatigue. **Methods:** In total, 56 pediatric cancer patients (≥7 and <23 years) who are 6 weeks post-acute cancer treatment will be included. Following baseline assessments (T0), patients are randomized (1:1) into an intervention (IG) or control group (CG). Both groups receive a brochure with suggested exercises and an activity tracker. The IG additionally receives a multimodal, partially supervised 12-week exercise program consisting of two training sessions per week, recommendations on daily physical activities and weekly movement goals. After the duration of 12 weeks of intervention (T1) and after a 12-week follow-up period (T2), assessments took place. **Results:** A total of 25 participants (45%) diagnosed with cancer (60% male, 12.5 ± 3.6 years, 40% leukemia, 24% Hodgkin lymphoma, and others) have been included, and recruitment is ongoing (25 September 2025). In total, 43 patients were eligible, and 25 (58%) agreed to take part in the study. Barriers and reasons for non-participation were mainly time and the need for detachment/separation from the clinic. Spiroergometry testing is feasible, and apart from the cognitive testing, all assessments were fully completed without any adverse events. **Conclusions:** The results will indicate the potential benefits of exercise as a simple way to prevent future long-term treatment effects and improve the general health state of these children.

### 8.20. Device-Based Monitoring of Physical Activity, Sedentary Behavior, and Sleep in Adult Survivors of Childhood Cancer: A National Czech Initiative with International Perspectives

Tomáš Vyhlídal ^1^, Jan Dygrýn ^1^, Tomáš Kepák ^2^, Jarmila Kruseová ^3^ and František Chmelík ^4^
^1^ Faculty of Physical Culture, Palacký University Olomouc, třída Míru 671/117, 771 11 Olomouc, Czech Republic
^2^ International Clinical Research Center, St. Anne’s University Hospital Brno
^3^ Department of Paediatric Haematology and Oncology, Motol University Hospital, Czech Republik
^4^ Faculty of Physical Culture, Palacký University Olomouc, Czech Republik

**Introduction:** Advances in pediatric oncology have led to survival rates exceeding 90% in many childhood cancers. With this success, attention has shifted to long-term survivorship care, where late effects of treatment—such as cardiometabolic complications, fatigue, neurocognitive decline, and impaired sleep—pose major challenges. Lifestyle-related behaviors, particularly sedentary behavior, physical activity, and sleep, represent modifiable factors with a substantial impact on quality of life and long-term health. Yet, systematic, objective monitoring of these behaviors in adult survivors of childhood cancer remains scarce. **Methods:** In the Czech Republic, we have established a unique research platform integrating clinical follow-up clinics for childhood cancer survivors with universities and patient organizations. Using device-based monitoring (accelerometry), we are conducting standardized assessments of sedentary behavior, physical activity, and sleep in adult survivors treated in childhood. Our projects involve both survivors of Hodgkin lymphoma and acute lymphoblastic leukemia, with ongoing expansion to survivors of CNS tumors and sarcomas. Recruitment is organized through specialized long-term follow-up clinics in Brno and Prague. This systematic approach provides high-quality, objective data that overcome the limitations of self-reports. **Results:** To our knowledge, the Czech cohort represents one of the few national-level initiatives combining accelerometer-based assessment of both movement behavior and sleep in long-term survivors of multiple childhood cancer diagnoses. This infrastructure provides a valuable opportunity for cross-country harmonization of methods, data sharing, and collaborative analyses. By joining efforts internationally, we can build larger datasets, identify risk profiles across populations, and develop evidence-based recommendations tailored to childhood cancer survivors transitioning into adulthood. **Conclusions:** National coordination in the Czech Republic demonstrates the feasibility and added value of systematic device-based monitoring of lifestyle behaviors in adult survivors of childhood cancer. We invite international partners to collaborate in data pooling, methodological harmonization, and intervention development to improve survivorship care and long-term health outcomes worldwide.

### 8.21. Feasibility of Implementing Physical Function Tests in Pediatric Hematopoietic Stem Cell Transplant Units: Preliminary Results of the HENKO Study

Eva Garrido-Rodríguez ^1^, Elena Higes-Núñez ^1^, Rocío Llorente-deSantiago ^2^, Belén Puente-Gómez ^2^, Eva SC-Ramos ^1,3^, Rodrigo Yagüe-Peñuelas ^1,3^, Natalia Yanguas-Casás ^1^, Elena Santana-Sosa ^1,3^, Laura González-Saiz ^1^, Félix Higes-Núñez ^1^, Alejandro Lucia ^1,3^ and Carmen Fiuza-Luces ^1^
^1^ Instituto de Investigación Sanitaria, Hospital Universitario 12 de Octubre, ‘Imas12’ (Madrid, Spain)
^2^ Fundación Unoentrecienmil (Madrid, Spain)
^3^ Universidad Europea de Madrid, Faculty of Sport Sciences (Madrid, Spain)

**Introduction:** Children with cancer undergoing hematopoietic stem cell transplantation (HSCT) are at high risk of physical and functional decline. The HENKO study evaluates the feasibility of implementing standardized physical function tests in routine clinical care within pediatric HSCT units. **Methods:** This is a prospective, non-controlled implementation study conducted in three pediatric HSCT units in Madrid, Spain. Clinical staff were invited to participate in HENKO by performing handgrip strength and functional capacity (5 min sit-to-stand [5-STS], 3 min step [3MST], and quick test) assessments of patients with pediatric cancer undergoing HSCT. Assessments were performed before HSCT (T-1), after hospital discharge (T1), and at follow-up (T2). Feasibility was determined through the completion rate of each test and the proportion performed by clinical staff. **Results:** A total of 43 patients have been included (47% female; mean age ± SD = 11 ± 4 years; 81.4% leukemias, 7% lymphomas, 7% retinoblastoma, 4.6% others). As of today, 85 clinicians have joined the HENKO project (84% female, 37 ± 12 years, 52% nurses, 23% physicians), of which 21 have actively participated. Handgrip strength was completed in 98% (T-1), 96% (T1), and 84% (T2) of cases, with nurse participation rates of 35%, 43%, and 46%, respectively. STS5 was performed in 100% (T-1), 96% (T1), and 83% (T2) of cases, with 43%, 43%, and 51% conducted by clinical personnel. The 3MST was completed by 93% (T-1), 86% (T1), and 76% (T2) of participants, with 42%, 50%, and 41% assessed by clinical staff. The Quick Test was achieved in 100% (T-1), 96% (T1), and 86% (T2) of cases, with participation rates of 43%, 45%, and 49% for the healthcare staff. **Conclusions:** These preliminary results suggest that physical function tests can be successfully implemented in the context of pediatric HSCT, supporting the integration of exercise programs into clinical practice. However, the exclusive involvement of clinical staff is not sufficient to ensure the feasibility of all tests. Future implementation strategies should involve exercise specialists to optimize feasibility and ensure high-quality assessments.

### 8.22. The Role of Physical Activity in Supporting Physical and Mental Health in Pediatric Cancer Patients and Survivors: A Systematic Review and Meta-Analysis

Ann Christin Schneider ^1^, Sofia Anzeneder ^1^, Lars Rehbein ^1^*,* Eva Brack ^2^*,* Mirko Schmidt ^1^ and Valentin Benzing ^1^
^1^ Institute of Sport Science, University of Bern, Switzerland
^2^ Department of Pediatric Hematology/Oncology, University Hospital Inselspital Bern, Switzerland

**Introduction:** Pediatric cancer patients and survivors are at risk for acute and long-term physical and mental health challenges due to their malignancy and treatment. Physical activity (PA) is increasingly recognized as a supportive intervention. This systematic review and meta-analysis investigates the effects of PA interventions on physical and mental health in pediatric cancer patients and survivors, including results from randomized controlled trials (RCTs) and clinical control trials (CCTs). **Methods:** A systematic search was conducted in APA PsycINFO, PubMed, SPORTDiscus, Web of Science, Embase, and Scopus. RCTs and CCTs investigating PA interventions in pediatric cancer patients and survivors were included if they reported physical, mental health, or quality of life outcomes. Two authors independently screened studies, extracted data, and assessed the risk of bias. A random-effects meta-analysis is ongoing, and the results will be incorporated upon completion. **Results:** Of 2936 articles screened, 43 RCTs and CCTs met the inclusion criteria, with 2315 participants aged 4–19 years. The included studies involved pediatric inpatients (*n* = 25), survivors (*n* = 15), or both (*n* = 3). The cancer type included multiple diagnoses (*n* = 26), solely leukemia (*n* = 12), and CNS tumors or neuroblastoma (*n* = 5). The PA interventions comprised different foci, including multimodal, endurance, strength, and free choice/sport games. Outcomes frequently assessed included cardiorespiratory fitness, strength, fatigue, depression, and quality of life. Positive effects were most observed for strength, fatigue, and self-efficacy (>50% of studies), with no reported negative effects. **Conclusions:** PA interventions have beneficial effects on pediatric cancer patients and survivors, yet intervention designs and outcomes remain heterogeneous. Research has mainly addressed physical and mental health, with limited attention to cognitive and motor abilities. High-quality RCTs and multivariate (network) meta-analyses are needed to identify the most effective approaches, strengthen the evidence base, and support integration of PA into standard care.

### 8.23. Precision-Based Exercise Protocols in Pediatric Oncology—The FORTEe Booklet

Francesca Lanfranconi ^1^, Eleonora Corti ^1^, Barbara Konda ^2^, William Zardo ^3^, Tommaso Moriggi ^1^, Linda Peli ^1^, Elias Dreismickenbecker ^4^, Marie A. Neu ^4^, Carmen Fiuza-Luces ^5^, Alejandro Lucía ^5^, Joerg Faber ^4^ and Adriana C. Balduzzi ^6,7^ on behalf of the FORTEe Consortium
^1^ Fondazione Monza e Brianza per il Bambino e la sua Mamma—Centro Maria Letizia Verga, Monza (Italy)
^2^ Forma 3D LTD, Ljubljana (Slovenia)
^3^ Pediatric Oncology Unit, Fondazione IRCCS Istituto Nazionale dei Tumori, Milan (Italy)
^4^ Universitätsmedizin der Johannes Gutenberg-Universität Mainz, Mainz (Germany)
^5^ Universidad Europea de Madrid Sau, Madrid (Spain)
^6^ Università degli Studi di Milano Bicocca, School of Medicine and Surgery, Milan (Italy)
^7^ Fondazione IRCCS San Gerardo dei Tintori, Monza (Italy)

**Introduction:** Children, adolescents, and young adults with cancer often experience reduced exercise tolerance, which negatively affects their physical and psychological abilities as well as their quality of life. Precision Exercise (PEx), tailored to the individual characteristics of each patient, can be an effective intervention to counteract the side effects of cancer treatments. The European research project FORTEe employs standardized exercise protocols compiled in a handbook available to pediatric oncology centers upon request. **Methods:** A multidisciplinary and multicenter team composed of sports medicine physicians and exercise professionals selected and adapted cardiorespiratory, strength, balance, and stretching exercises. The handbook also includes protocols for personalizing physical activity according to specific clinical conditions, as well as motivational strategies to make exercise more engaging and inclusive. **Results:** A total of 90 exercises were illustrated, each with its corresponding adapted version. The first edition of the handbook was released in May 2025 during the SIOP Europe Congress in Budapest. A total of 237 copies were distributed across 30 European countries, as well as in the United States, South America, and New Zealand. Pediatric oncologists, nurses, researchers, exercise professionals, and family associations received the handbook. **Conclusions:** The handbook developed within the FORTEe project represents an innovative tool for promoting PEx in care centers for pediatric oncology patients. Thanks to its clear structure and adaptability to various clinical conditions, it has been widely recognized by the scientific community and parent associations.

### 8.24. From Evidence to Everyday Practice: Implementing Physical Activity in Pediatric Oncology Through the KiKli Fit Project

Lars Rehbein ^1^, Ann Christin Schneider ^1^, Lisa Hillebrecht ^2^, Christina Schindera ^3^, Amika Singh ^4^, Eva Katharina Brack ^2,a^ and Valentin Benzing ^1^
^1^ Institute of Sport Science, University of Bern, Switzerland
^2^ Department of Pediatric Hematology/Oncology, University Hospital Inselspital Bern, Switzerland
^3^ Department of Pediatric Hematology/Oncology, University Children’s Hospital Basel, Basel, Switzerland
^4^ Mulier Instituut, Herculesplein 269, 3584AA Utrecht, The Netherlands
^a^ Both authors share senior authorship

**Introduction:** Growing evidence in pediatric oncology highlights the benefits of physical activity (PA) during and after cancer treatment toward physical and mental health. However, systematic promotion of PA during cancer therapy remains rare in Switzerland. To address this gap, the KiKli Fit project was developed, offering supervised PA therapy at the University Hospital of Bern. The aim was to evaluate its integration into clinical practice, assess acceptance among stakeholders, and explore the barriers and facilitators crucial for successful implementation. **Methods:** The Consolidated Framework for Implementation Research (CFIR) was used to structure, develop, implement, and evaluate the KiKli Fit project. A participatory approach, including a needs analysis and focus group, guided the process. Twenty-three interviews were conducted with patients, parents, healthcare professionals, and implementation coordinators. These interviews explored attitudes, barriers, and facilitators of implementation in pediatric oncology and were analyzed using content analysis. The PA sessions were documented to assess implementation outcomes, including reach, feasibility, and fidelity. **Results:** The findings indicate broad acceptance among patients, parents, and healthcare professionals. Patients reported positive experiences, including improved well-being resulting from participation. Healthcare professionals highlighted the successful integration of the project into clinical practice and the benefits to patients’ mental health. The participatory approach, stakeholder communication, and strategies to address limited resources were the main facilitators. Implementation outcomes indicated that PA sessions reached a large proportion of patients, although medical constraints or treatment schedules limited participation. **Conclusions:** High acceptance among all stakeholders underscores the importance of PA therapy for improving treatment quality. Future efforts should address barriers such as limited integration into routine care and challenges related to participation, while acknowledging that full inclusion may not always be feasible. Long-term sustainability will further depend on securing stable funding to ensure continued implementation and expansion as a core element of pediatric oncology care.

### 8.25. SHAPED—Health Behaviour Interventions to Support Children and Young People Post-Cancer Treatment: A Rapid Scoping Review

Dr Esther Carter ^1^, Dr Martin Lamb ^1^, Debbi Rowley ^2^, Sarah Massey ^3^ and Dr Liam Humphreys ^1^
^1^ Advanced Wellbeing Research Centre, School of Sport and Physical Activity, Sheffield Hallam University, Sheffield, United Kingdom
^2^ Sheffield Children’s Foundation Trust, Sheffield, United Kingdom
^3^ Illingworth Library, Sheffield Children’s Foundation Trust

**Introduction:** Children and young people (C&YPs) who have undergone cancer treatment may exhibit decreased physical activity (PA) levels, increased risk of malnutrition ranging from undernutrition to obesity and worsened mental health. Despite the reported benefits of promoting health behaviour in C&YPs post-treatment to improve physical and mental health and reduce the reoccurrence of secondary cancers, support is not offered as part of routine paediatric care. It is unclear what support is currently available for C&YPs post-cancer treatment to encourage health behaviours. This review will identify health behaviour interventions that are currently being delivered and their key components. **Methods:** A rapid scoping review will be conducted in accordance with JBI guidelines to allow a more flexible, broad exploration of emerging areas of research. Academic databases alongside grey literature will be searched to identify interventions promoting health behaviours in C&YPs (under 18) post-cancer treatment. The Taxonomy of Behaviour Change Techniques will be used to identify active components of interventions. Data screening and extraction will be managed in Covidence, and thematic analysis of findings will be conducted using NVivo independently by two authors. **Results:** This review will allow us to identify: (1) types of support interventions available; (2) key characteristics and design components of interventions; (3) use of behaviour change theories; (4) intervention outcomes; and (5) limitations and future learnings. **Conclusions:** Learnings will be used to inform the co-design of a future multi-modal healthy behaviours programme for C&YPs who have completed treatment for cancer. Specifically, understanding the BCTs currently in use will allow further research into whether these are appropriate for C&YPs.

### 8.26. SHAPED: Service Development of a Healthy BehAviours ProgrammE for ChilDren and Young People Post Cancer Treatment

Dr Martin Lamb ^1^, Dr Esther Carter ^1^, Debbi Rowley ^2^ and Dr Liam Humphreys ^1^
^1^ Advanced Wellbeing Research Centre, School of Sport and Physical Activity, Sheffield Hallam University, Sheffield, United Kingdom
^2^ Sheffield Children’s Foundation Trust, Sheffield, United Kingdom

**Introduction:** The use of complex treatments (e.g., chemotherapy,) has led to paediatric cancer survival rates doubling since the 1970s. However, cancer treatment for children and young people (C&YPs) can negatively impact health and future chronic conditions. Interventions to support physical activity (PA) and nutrition in C&YPs can positively influence physical and mental health. Nevertheless, some C&YPs and their families report insufficient support post-treatment. The aim of SHAPED is to co-design a multi-modal health behaviours programme for C&YPs aged 0–19 years, who have completed their cancer treatment. The presentation will provide an overview of the SHAPED methodology. **Methods:** The project consists of five work packages (WPs): WP1—Scoping review of existing post-cancer health behaviours programmes for C&YPs; WP2—Online survey and interviews with healthcare professionals (HCPs) to understand current experiences and future preferences for post-cancer support for C&YPs; WP3—Explore the preferences for and lived experiences of C&YPs and their families engaging with health behaviour support through creative and traditional (e.g., interviews, online surveys) research approaches; WP4—Interviews with community stakeholders with experience in supporting C&YPs during and after cancer treatment; WP5—The findings from WPs 1–4 will then inform three workshops to be held with C&YPs, their families, HCPs, and community stakeholders to co-design a new support programme. **Results:** As the project is ongoing, an overview of the methodology will be discussed alongside insights on progress, preliminary findings, and challenges in delivery so far. **Conclusions:** The SHAPED project will lead to the co-design of a new, multimodal support programme for C&YP post-cancer treatment to improve their mental and physical health.

### 8.27. Frankfurt Exercise and Nutritional Therapy Registry in Pediatric Oncology: Study Protocol

Mirjam Dieckelmann ^1^, Aleksandar Tomaskovic ^2^, Vera Blümer ^2^ and Sandra Krattenmacher ^2^
^1^ Frankfurt University Medicine, Theodor-Stern-Kai 7, 60590 Frankfurt, Germany
^2^ Department of Pediatrics, Frankfurt University Medicine, Frankfurt, Germany

**Introduction:** Exercise and nutritional therapy are recommended throughout pediatric cancer treatment and survivorship, yet are not part of standard care and lack robust real-world data. This limits systematic evaluation and cross-institutional learning. This project aims to establish a local, quality registry to systematically collect routine data on therapy delivery and outcomes related to physical and nutritional status in children, adolescents, and young adults with cancer. **Methods:** This non-controlled, local registry will include anonymized retrospective clinical data from sports therapists at the Department of Pediatrics, University Hospital Frankfurt (from June 2023), with prospective data collection starting January 2026 for three years. All eligible pediatric oncology patients receiving exercise and/or nutritional therapy per standard operating procedures and providing consent will be included. Data will be collected at five defined time points along the care continuum: at diagnosis, at the end of acute treatment, at six months post-catheter explant, at twelve (low-risk) or eighteen (high-risk) months post-catheter explant, and at five years post-catheter explant. Variables include nutritional status, motor skills, functional mobility, psychosocial health, and physical performance, assessed through electronic health records, therapist evaluations, and patient questionnaires. **Results (Expected):** The registry is designed to align with clinical workflows, enabling comprehensive documentation of routine care. It will support identification of key intervention points across tumor types, age groups, and treatment intensities. The registry will allow prevalence estimates of malnutrition and physical deconditioning for the Frankfurt pediatric oncology population. **Conclusions:** We will present the registry protocol, identify early technical and organizational challenges, and critically discuss its role in driving data-based quality improvement in pediatric exercise oncology.

### 8.28. Towards Developing a Digital Tool to Navigate the Paediatric Sports Therapy Journey

Kimberly-Annalena Munjal ^1^, Aleksandar Tomaskovic ^2^, Vera Blümer ^2^, Sören Aguirre-Reid ^1^, Frank Kammer ^2^, Markus Siepermann ^2^ and Mirjam Dieckelmann ^3^
^1^ Technische Hochschule Mittelhessen
^2^ Department of Pediatrics, Frankfurt University Medicine
^3^ Frankfurt University Medicine, Theodor-Stern-Kai 7, 60590 Frankfurt, Germany

**Introduction**: Sports therapy plays a vital role in the improvement of health and quality of life for children and adolescents with cancer. Still, resources for therapy, including time and personnel, are often limited. At Frankfurt University Hospital, therapy planning, documentation, and time scheduling are largely done manually—placing a significant administrative burden on therapists and limiting continuity across the care pathway. This project aims to develop a user-centred mobile app to support sports therapists throughout the therapy journey, from initial assessment to documenting progress and outcomes. **Methods**: Using a user-centred design approach, we conducted a requirement analysis informed by clinical workflows. For the prototype development, we integrated multiple feedback loops with the therapists to ensure the tool has an optimal task fit. Moreover, a systematic literature review supports the development of suitable gamification elements for the patients. This approach guided the development of a gamified mobile app prototype that facilitates therapy documentation, session planning, motivational communication, and structured data sharing with patients and families. **Results**: We will present a clickable gamified mobile app prototype, demonstrating core features and therapist–patient interaction points. Designed specifically for the clinical paediatric oncology setting, the app offers an intuitive interface for therapists to display and record therapy data on the go. The app will track patient engagement, provide gamification elements to enable families to stay informed and motivated, and personalise therapy plans. **Conclusions**: A digital companion app has the potential to reduce administrative burden for therapists while improving patient engagement and the continuity of care through accessible, shared therapy data. Challenges for further development, user feedback and future steps towards implementation will be critically discussed.

### 8.29. Feasibility and Impact of an Exercise Program for Children with Leukemia and Lymphoma During the First Month of Treatment: A Protocol for a Clinical Trial in Uruguay

Analía Acuña ^1^, Mariana Gómez-García ^1^, Verónica Búa ^1^, Paloma Amarillo ^2,3^ and Carolina Chamorro Viña ^4,5^
^1^ Instituto Superior de Educación Física, Universidad de la República, Montevideo, Uruguay
^2^ Facultad de Medicina, Universidad de la República, Montevideo, Uruguay
^3^ Fundación Pérez Scremini, Montevideo, Uruguay
^4^ University of Calgary, Faculty of Kinesiology, Calgary, Canada
^5^ Kids Cancer Care Foundation of Alberta, Calgary, Canada

**Introduction:** Children undergoing treatment for leukemia or lymphoma often experience side effects that reduce quality of life. Growing evidence indicates that supervised physical activity (PA) is safe and beneficial even during acute treatment, helping to ameliorate treatment-related side effects. However, such interventions are not yet implemented in Uruguay. **Objectives**: This study aims to determine the feasibility and potential benefit of an exercise program (EP) during the first month of treatment in children and adolescents newly diagnosed with leukemia or lymphoma. **Methods:** This two-arm trial will recruit 30 children (4–18 years) newly diagnosed with leukemia or lymphoma. Participants will be randomly assigned to: Arm A—five sessions/week, including three days of aerobic exercise (2 × 10–15 min, 40–60% HRR/RPE 3–6), two days of strength training (8–10 exercises, 1 × 8–12 reps, progressive load), plus Proprioceptive Neuromuscular Facilitation flexibility training twice/week; or Arm B—two sessions/week of strength and flexibility training (same as Arm A, without aerobic exercise). Participants will complete a 30-day in-hospital intervention, followed by the option to join a 60-day mixed home/hospital follow-up. **Results:** This study looks to assess feasibility (recruitment, adherence), motor development (MOON test), functional capacity (TUG), quality of life and fatigue (PedsQL cancer module and fatigue). Adverse events will be recorded. Assessments will occur at baseline and day 31. Differences between arms will be compared using SPSS Version 32, applying the Mann–Whitney U test where appropriate. **Conclusions:** This is the first study of this type of EP in Uruguay, providing evidence for the feasibility of exercise as adjunct therapy during treatment. A subsequent multi-center RCT will evaluate effectiveness in pediatric oncology across broader diagnoses, aiming to incorporate exercise programs into standard care for children with cancer in Uruguay.

### 8.30. “I Can’t Tell if He’s Unwell…or Just Bored”: An Enriched Ward Environment as the Missing Ingredient to Improving Both Physical and Daily Activity Levels in Paediatric Oncology

Vicky King ^1^ and Rebecca Tapley ^2^
^1^ Brainbow, Neuro-Oncology rehabilitation, Cambridge University Hospitals NHS Foundation Trust, Hills Road, CB2 0QQ Cambridge, United Kingdom
^2^ Brainbow, Neuro-oncology, Cambridge University Hospitals NHS Foundation Trust

**Introduction:** Despite an awareness that maintaining activity levels is important to reduce the late effects of cancer treatment, children in oncology wards remain the most sedentary of inpatients. The reasons for this are multifactorial; however, the current literature is mainly focused on physical activity and exercise, not other forms of daily activity (such as self-care, education or leisure), and within adults rather than children. **Methods:** Patients, families and staff in a paediatric oncology ward completed questionnaires to gather data around levels of physical and daily activity at home compared to when at the ward. They were asked for their opinions on the importance of and barriers and facilitators to activity whilst an inpatient. **Results:** Despite rating maintaining activity as important, all patients, families and staff report a drop in both physical and daily activity levels as an inpatient compared to when at home, which included basic self-care tasks as well as pleasurable daily activities such as getting fresh air, time with family/friends and bedtime routines. Analysis of the qualitative data is suggestive of environmental factors within the ward (lack of opportunity, role modelling and culture) as the main barrier, rather than patient or physiological issues. **Conclusions:** Despite the known benefits of activity for children undergoing cancer treatment, our data shows a startling drop in both physical and daily activity (washing, dressing, etc.) when children are admitted for inpatient treatment, with patients, parents and staff citing environmental factors as a main cause for the decline. Both physical and daily activity should therefore be targeted when aiming to improve overall activity, with ward-wide work at a universal level (such as education, modelling and provision of activity) to facilitate a beneficial ward environment, alongside the more traditional specialist/targeted intervention on an individual basis.

### 8.31. Moving Forward: Feasibility of a Structured Exercise Program in Pediatric Oncology

Joana Rebelo ^1,2^, Janine Coelho ^1,2^, Rita Pires ^1,2,3^, Sónia Sampaio ^1,2^, Nélia Gaspar ^1,2^ and Maria do Bom-Sucesso ^1,2,4^
^1^ Pediatric Oncology Department, ULS São João, Porto, Portugal
^2^ ERN PaedCan, European Reference Network for Paediatric Cancer
^3^ Superior School of Nursing, University of Porto, Portugal
^4^ Medical Faculty, University of Porto, Portugal

**Introduction:** Pediatric cancer and its treatments are frequently associated with fatigue, sarcopenia, musculoskeletal complications, impaired cardiorespiratory fitness, and loss of autonomy. Hospitalization further exacerbates inactivity, accelerating deconditioning and reducing quality of life. Evidence shows that tailored exercise interventions are safe, feasible, and beneficial. However, their integration into clinical care remains limited, particularly in our country, where no structured hospital-based programs currently exist. This project aims to develop and implement a structured exercise program within a Portuguese pediatric oncology reference center dedicated to solid tumors, evaluating its feasibility in routine clinical practice. **Methods:** The program will include two components: individualized bedside sessions during inpatient stays, adapted to clinical status and using simple, hygienic equipment; and group classes in the outpatient day-care unit, delivered in a playful format to foster socialization, motivation, and peer support. Exercises and materials will be adapted to hospital spaces, without requiring dedicated exercise areas, and will rely on lightweight, hygienic equipment occupying minimal space. A multidisciplinary team of physicians, nurses, and exercise professionals will develop standardized, evidence-based protocols and support materials, including an individual logbook, illustrated plans, and instructional videos. Feasibility outcomes will include recruitment and adherence rates, safety (adverse events), acceptability by patients and families, and integration within clinical workflows. Validated functional tests and patient-reported outcome measures will assess preliminary effectiveness. **Results:** High adherence and acceptability are anticipated, with minimal adverse events. Improvements are expected in physical fitness, emotional well-being, and motivation. **Conclusions:** This structured exercise program represents an innovative, child- and family-centered intervention that is feasible in the Portuguese pediatric oncology context. It has strong potential for integration into daily clinical practice, contributing to improved care and health outcomes.

### 8.32. Availability and Accessibility to Exercise-Related Health Information Resources (ErHIRs) for Children with Cancer—A Scoping Review

Nivedita Prabhu ^1^, Vishrutha Vishwanath ^1^, Tapasya Shetty ^1^, Veena Bhat ^1^ and Vasudeva Bhat K. ^2^
^1^ Physiotherapy, Manipal College of Health Professions, Manipal Academy of Higher Education, 576104 Manipal, Karnataka, India
^2^ Pediatric Hematology and Oncology, Manipal Comprehensive Cancer Care Centre, Kasturba Medical College, Manipal Academy of Higher Education, 576104 Manipal, Karnataka, India

**Introduction**: Regular exercise is essential in regulating the well-being of children with cancer. Global access to reliable exercise-related health information resources (ErHIRs) is crucial in empowering them and their caregivers to make informed decisions related to getting fitter. These resources would provide evidence-based recommendations and guidance, ensuring that children with cancer can safely engage in exercise programs to support their recovery. We aimed to identify, collate and map the existing published literature related to the availability and accessibility of exercise-related health information resources for children with cancer. **Methods**: This review was registered under the Open Science Framework as per the Population–Concept–Context (PCC) framework by Arksey and O’Malley, using the six-stage methodological process. The population selected was children with cancer, the concept was ErHIRs, and the context was related to the availability and accessibility of these resources. A focused research question was established, followed by a preliminary literature search. The search strategy included the following databases (PubMed, Cochrane Central, CINAHL, Scopus) using keywords (children with cancer, caregivers, family, exercise, health information resources, health literacy, digital health, exercise manual). Resources for adults with cancer, non-exercise-related resources, and unpublished data were excluded. The Preferred Reporting Items for Systematic Reviews and Meta-Analyses extension for Scoping Reviews (PRISMA-ScR) checklist was used to ensure transparency and quality. Records were included on a consensus of 90%. **Results**: Nine records were obtained after screening for title/abstract; full texts, including four resources available in e-formats; and papers providing details of different cancers, specific exercises, precautions and alternatives. The collected chapters focused on aerobic exercise, resistance training, flexibility, balance, coordination and recreation. Accessibility through the internet, conferences, symposiums, and patients themselves was observed. **Conclusions**: Easy accessibility and availability of ErHIRs provide better guidance to children with cancer and their families, thereby enhancing their levels of physical fitness and mental wellbeing.

### 8.33. The Formation of a Multidisciplinary, Clinical Exercise Program for Childhood, Adolescent, and Young Adult Cancer Survivors in the United States

Ellen K. Chang, MD, MS ^1,2^, William Hardie, MD ^1,3^, Christin Zwolski, DPT, PhD ^1,4,5,6^, Jennifer Bernstein, DPT ^4^, Robert Hyde, DPT ^4^, Caleb Parsons, MS ^7^, Iesha Gover, MS ^3^ and Adam W. Powell, MD, MS ^1,7^
^1^ Department of Pediatrics, University of Cincinnati College of Medicine, Cincinnati, OH, USA
^2^ Division of Oncology, Cancer and Blood Diseases Institute, Cincinnati Children’s Hospital Medical Center, Cincinnati, OH, USA
^3^ Division of Pulmonology, Cincinnati Children’s Hospital Medical Center, Cincinnati, OH, USA
^4^ Division of Occupational Therapy and Physical Therapy, Cincinnati Children’s Hospital Medical Center, Cincinnati, OH, USA
^5^ Division of Patient Services Research, Cincinnati Children’s Hospital Medical Center, Cincinnati, OH, USA
^6^ Division of Sports Medicine, Cincinnati Children’s Hospital Medical Center, Cincinnati, OH, USA
^7^ The Heart Institute, Cincinnati Children’s Hospital Medical Center, Cincinnati, OH, USA

**Introduction**: Exercise therapy has been shown to mitigate short- and long-term toxicities of cancer therapies, improve functional capacity, decrease the risk of disease recurrence and prolong life in cancer survivors. Unfortunately, pediatric exercise therapy programs have largely been limited to research initiatives in the United States. This has resulted in a vast underutilization of these services, depriving this population of clinically impactful treatment. **Methods**: We discuss the processes and general framework for a novel, clinically based and funded exercise program for childhood, adolescent, and young adult (CAYA) cancer survivors in the United States. **Results**: The Cardiorespiratory Program for Wellness (CRP-Well) at Cincinnati Children’s Hospital Medical Center (CCHMC) is a comprehensive and individualized exercise program for CAYA cancer survivors, developed by a multi-disciplinary team. The care delivery model for this program is based on our existing model for cardiac rehabilitation, which includes up to 36 in-person and virtual exercise therapy sessions with pre- and post-program standardized assessments. Data from pre-program cardiopulmonary exercise testing and physical therapy evaluation guide the individualization of programming. Longitudinal assessment of primary outcome measures includes clinical and molecular markers of aging and cardiopulmonary dysfunction, exercise adherence, program acceptability, and cost-effectiveness. Through the utilization of clinical billing structures in existing therapy models, this program has demonstrated financial viability in its pilot phase, serving as a framework for further clinical growth. **Conclusions**: Through integrated efforts led by a multi-disciplinary team at CCHMC, CRP-Well was established to provide an unmatched level of expertise and services in the field of pediatric exercise medicine to impact the lives of CAYA cancer survivors. Future goals of this program are to provide an infrastructure using this financially sustainable model for expansion to other pediatric populations with chronic diseases and provide reproducibility for other institutions nationally.

### 8.34. A Virtually Delivered Home-Based Exercise Intervention on Cognitive Impairment and Gut Microbiome in Adolescent and Young Adult (AYA) Brain Tumor Survivors

Maria C. Swartz ^1^, Keri Schadler ^2^, Tricia King ^3^, Avery Griffen ^3^, Ashley Rajan ^2^, Caitlin Webster ^3^, Theresa Honey ^2^, Alison Vargo ^2^, Jenny Blevins ^2^, Emma Chow ^3^, Emily LaVoy ^4^, Nathan Parker ^5^, Shiao-Pei Weathers ^2^, Tobey MacDonlad, MD ^6^, Jinbing Bai ^3^
^1^ University of Texas Medical Branch, Houston, TX, USA
^2^ MD Anderson Cancer Center, Houston, TX, USA
^3^ Nell Hodgson Woodrufff School of Nursing, Emory University, Atlanta, GA, USA
^4^ University of Houston, Houston, TX, USA
^5^ Moffit Cancer Center, Tampa, FL, USA
^6^ School of Medicine, Emory University, Children’s Healthcare of Atlanta, Atlanta, GA, USA

**Background and Aims**: Up to 45% of AYA brain tumor survivors experience cancer-related cognitive impairment (CRCI) affecting processing speed, attention, working memory, and executive function—domains critical for quality of life (QOL). Moderate-intensity exercise can improve cognitive function, potentially through changing the gut microbiome. Few randomized controlled trials (RCTs) have examined virtually delivered exercise interventions on CRCI among AYA brain tumor survivors. The RISE trial (NCT06799481) aims to evaluate feasibility, cognitive and physical activity outcomes, and gut microbiome changes from a 12-week virtually delivered exercise intervention with behavioral coaching. **Methods**: RISE is a two-arm pilot RCT of 40 AYA brain tumor survivors with CRCI enrolled at Emory University and MD Anderson Cancer Center. Controls receive a Fitbit and 18 wellness calls. Intervention participants receive a Fitbit, 18 virtual exercise coaching sessions, and self-led sessions via the Physitrack telehealth app for 12 weeks. Assessments at baseline, 6, 12 (primary), and 18 weeks are conducted to measure cognitive function, physical activity, gut microbiome composition, and QOL. **Results**: A total of 13 survivors have been enrolled and randomized (2.5% of those approached by email or cold call have consented). Retention was 92.3% (12/13), with one withdrawal due to time conflicts. All participants completed T0 and are continuing with no adverse events reported. Among three participants who completed 12 weeks, adherence was 100%. Key implementation learning: scheduling flexibility outside work and childcare hours is critical, and there is a need to adapt exercise equipment to accommodate functional limitations among brain tumor survivors. **Conclusions**: This ongoing pilot demonstrates that virtual exercise can be safely delivered to AYA brain tumor survivors. Adherence and retention rates indicate strong patient engagement. Findings will inform a larger RCT to determine the efficacy of this scalable, mechanism-informed exercise intervention to mitigate CRCI.

## Data Availability

No new data were created or analyzed in this study. Data sharing is not applicable to this article.

